# Heterogeneous Evolution of Breast Cancer Cells—An Endogenous Molecular-Cellular Network Study

**DOI:** 10.3390/biology13080564

**Published:** 2024-07-26

**Authors:** Tianqi Li, Yong-Cong Chen, Ping Ao

**Affiliations:** 1 Center for Quantitative Life Sciences & Physics Department, Shanghai University, Shanghai 200444, China; tancygd@shu.edu.cn; 2 School of Biomedical Engineering, Sichuan University, Chengdu 610065, China; aoping@sjtu.edu.cn

**Keywords:** breast cancer, cell subtype, molecular regulatory network, systems biology

## Abstract

**Simple Summary:**

The classification of breast cancer has been a complex subject, with diverse origins of normal breast epithelial cells and evolutionary pathways contributing to its extensive heterogeneity, which further complicates the treatment process. We developed a dynamic network model to simulate the emergence of different cellular states and explore the underlying biological mechanisms. This model revealed cellular states that align with four recognized molecular subtypes of breast cancer. It also identified feedback loops that either reinforce or facilitate phenotypic transitions, and allowed us to trace these transitions through the model’s topological structure. This approach provides new insights into the mechanisms driving breast cancer heterogeneity and may benefit the development of more targeted therapeutic strategies.

**Abstract:**

Breast cancer heterogeneity presents a significant challenge in clinical therapy, such as over-treatment and drug resistance. These challenges are largely due to its obscure normal epithelial origins, evolutionary stability, and transitions on the cancer subtypes. This study aims to elucidate the cellular emergence and maintenance of heterogeneous breast cancer via quantitative bio-process modeling, with potential benefit to therapeutic strategies for the disease. An endogenous molecular–cellular hypothesis posits that both pathological and physiological states are phenotypes evolved from and shaped by interactions among a number of conserved modules and cellular factors within a biological network. We hereby developed a model of core endogenous network for breast cancer in accordance with the theory, quantifying its intrinsic dynamic properties with dynamic modeling. The model spontaneously generates cell states that align with molecular classifications at both the molecular and modular level, replicating four widely recognized molecular subtypes of the cancer and validating against data extracted from the TCGA database. Further analysis shows that topologically, a singular progression gateway from normal breast cells to cancerous states is identified as the Luminal A-type breast cancer. Activated positive feedback loops are found to stabilize cellular states, while negative feedback loops facilitate state transitions. Overall, more routes are revealed on the cellular transition between stable states, and a traceable count explains the origin of breast cancer heterogeneity. Ultimately, the research intended to strength the search for therapeutic targets.

## 1. Introduction

The prevalence of breast cancer (BC) has been rising annually at a rate of approximately 0.5%, surpassing lung cancer to become the most common cancer globally since 2021 [1,2]. Despite the notably high five-year survival rate following treatment, BC persistently remains a primary cause of cancer-related fatalities among women [3,4]. A significant obstacle in developing effective treatment strategies is the inherent heterogeneity of the disease, which prominently affects clinical diagnosis and treatment resistance [5,6]. Over the past two decades, with increasing recognition and accommodation of the disease’s inherent heterogeneity, therapeutic strategies for BC have progressively shifted towards biologically targeted therapies and treatment de-escalation [5]. Both approaches are distinguished by molecular subtypes of the cancer, with an aim to minimize adverse effects [7,8,9]. Though the paradigm in BC treatment is transitioning towards personalization, the precision medication demands high costs and advanced expertise from physicians [8,10]. The exploration of the origin cells of breast cancer and the plasticity factors driving different BC subtypes remain critical to allow our understanding of tumor heterogeneity and the design of therapeutic interventions [11].

The heterogeneity present in BC is complex, exhibiting high diversity both intertumorally and intratumorally [12]. Based on distinctions in morphology and histology, the WHO Breast Tumour Classification Guidelines, updated in 2019, categorize the disease into numerous histological categories, including but not limited to invasive lobular and tubular carcinomas [13]. This classification is based on the similarity in morphology and anatomical location between the tumor cells and normal cells. But how accurate are they? The histological classification follows an exclusionary approach, where only a small portion of cancer cells with sufficiently clear morphology can be adequately classified into a proper subtype. Meanwhile, the majority that fail to match a confirmed cell morphology are instead classified as a non-specific type of invasive ductal carcinoma, accounting for a stark 50–80% of cases [14]. However, this intuitive performance suggests that BC originates from the corresponding types of normal epithelial cells [15], and hypothesizes that heterogeneity arises from the dynamic nature of the breast organ which allows multiple types of normal breast epithelial cells to coexist in the tissue [16,17,18].

The concept of molecular subtypes emerged with the maturation of sequencing technology and molecular detection methods such as immunohistochemistry [19]. Proposed by Perou and colleagues in 2000, it divided BC into five molecular subtypes based on gene expression clustering of microarrays, each characterized by distinctive expression and growth rates of estrogen receptor (ER), progesterone receptor (PR), and human epidermal growth factor receptor2 (HER2) [20]. Among them, a “normal-like” subtype of BC was so named because its expression spectrum resembles that of normal mammary cells. But it could not be subsequently reproduced, rendering the result as normal cells mixed into the sample [21,22]. The alternative classification has since significantly contributed to the improvement of treatment and prognosis of the cancer. Nonetheless, the underlying criteria are often criticized for relying too much on a purely descriptive method of supervised data clustering [14].

Proponents of molecular subtyping argue that the phenotypes of breast cancer reflect the phenotypes of their origin cells based on similarities in gene profiles, further supporting the molecular subtypes as disease entities [23]. However, even if a tumor has a protein expression profile similar to that of some normal cells, does this prove that the tumor originated from cells with this differentiation profile [24]? It is worth noting though that the profiles of luminal progenitor cells are more similar to those from basal-like BC. On the other hand, most of invasive BCs originate from terminal duct-lobular units (TDLU), as indicated by numerous experiments [8,25,26]. There are many types of cancer that do not reflect their micro-anatomical origin but manifest in cellular morphology [27]. Moreover, the genetic similarities between low-grade ER+ ductal carcinoma and lobular carcinoma make the ER+ and ER− breast cancers different evolutionary stepping lineages not distinguished by histology [28].

Despite the vast amount of data provided by omics analyses and single-cell technologies, they have not resolved the perplexing controversies regarding the origins and development of BC heterogeneity [29]. Such complexity of mammary gland development leads some scholars to claim that “defining the cell-of-origin and evolutionary pathway of a breast cancer in humans is a nearly impossible task, as we are rarely able to diagnose tumors at their earliest stages and follow their molecular evolution” [12]. To address this challenge, we employed a dynamic network approach which integrates the genetic hierarchical structure within biological systems and efficiently simulates the spontaneous evolutionary process of cells from a dynamic perspective. In contrast to the earlier methods that focus purely on molecular data, the endogenous network approach characterizes cellular behaviors as well as traces their evolutionary origins [30]. This quantifiable hypothesis, proposed from the systems biology perspective, posits that cancer is an inherent state shaped by its underlying endogenous molecular–cellular network [31]. This method has proven effective in explaining diseases such as prostate cancer, colorectal cancer, early pancreatic development, and other cellular properties [32,33,34].

In this study, we retrieved characteristics of BC and constructed a core endogenous network composed of a set of key factors (genes/molecules) and their interactions. The system was then analyzed via nonlinear stochastic dynamics to quantify in particular the cellular states or distinct biological phenotypes by the expression of these factors. These states manifest as stable attractors on the endogenous network, validated with the clinical data of BC samples in the TCGA database (including healthy samples). Thirteen cellular states, with four inherent molecular subtypes of BC, as well as normal and apoptotic cell types, spontaneously emerged in the dynamics. This suggests that the working network is potentially valid for studying both normal breast tissue and BC. It also allowed us to trace the evolutionary paths of steady states through saddle points to reveal the topology of occurrence and pathways of heterogeneity, laying a groundwork for exploring the origin cells of BC and therapeutic treatment.

## 2. Materials and Methods

### 2.1. Biological Hypothesis and Principles of Network Construction

The foundation which we used to articulate the dynamics of breast cancer lays on the endogenous molecular–cellular network mentioned above. Underlying the approach is a hypothesis that biological evolution gradually leads an organism to develop a core regulatory network, a minimal ensemble of endogenous factors (genes/molecules/modules) essential for maintaining functional efficacy [31]. The expression or activation of these factors are critical for the survival of the biological system. Due to evolution-conserved characteristics, there is little chance of any major modification of the essential structure of this network [30]. The states of the network evolve over time under nonlinear, mutual interactions among the factors. The system manifests itself in a high-dimensional, nonlinear, stochastic/dynamical platform, enabling comprehensive and quantitative portrayal of an intricate molecular–cellular network.

In the construction phase, we adhere to the fundamental principles of biological modularity and hierarchical structuring, positing that decisions within biological systems are autonomously determined by independently executable functional modules and the crosstalk between them. The essential modules incorporated into the network must ensure the execution of essential physiological functions and fulfill the research objectives. To demonstrate the core regulatory mechanisms of BC mechanistically, the model considers general cancer traits and integrates fundamental features of breast development and cancer [35,36]. This encompasses modules related to the cell cycle, cell apoptosis, inflammation, metabolism, angiogenesis, adhesion, and BC specific factors, each backed by comprehensive research [37].

The components of these modules—including DNA, proteins, and mRNA—participate in a complex network inter-relationship. The interactions among these factors are meticulously investigated and sourced from the literature, supported by well-documented gene regulatory networks and convincing molecular biological experiments. Obtaining complete in vivo parameters for the interactions is clearly challenging. We therefore employed a coarse-grained abstraction, categorizing them as either ‘activating’ or ‘inhibiting’ [31]. At the core level, we took on a close system where interaction/information is non-unidirectional, i.e., no element is exclusively upstream or downstream. Each factor acts on and is influenced by other factors on the network.

### 2.2. Quantitative Simulation of Endogenous Molecular–Cellular Network

In the core endogenous network, a node represents a distinct key factor, which can be regarded as a dimension in phase space. The level and variations of its relative expression are co-determined by both deterministic regulation and stochastic influences. Consequently, in a network model of N nodes, the temporal variation for the ith factor can be described by a stochastic differential equation:

(1) $$\frac{dx_{i}}{dt}=g_{i}\left(x,\alpha \right)-\frac{x_{i}}{\tau_{i}}+\zeta_{i}\left(x,t\right),$$

where the transpose of the column x vector $x^{T}=\left(x_{1},x_{2},\ldots ,x_{N}\right)$ represents the expression level or the activity of the entire network at a given time. The term $g_{i}\left(x,\alpha \right)-x_{i}/\tau_{i}$ denotes the deterministic part of the equation (referred to as f hereafter), simulating the rate of change in the factor’s concentration or activity. $g_{i}\left(x,\alpha \right)$ (with a generic/unspecified parameter α) indicates the production rate under influence from others, while $\tau_{i}$ sets a timescale of degradation for the factor. Assuming cellular division or apoptosis are the primary influencing factors, $\tau_{i}$ is normalized to unity for the sake of simplicity. The term $\zeta_{i}$ suggests a multiplicative Gaussian white noise of zero mean, reflecting the influences such as accumulated intracellular fluctuations or environmental changes. The type of equations can be handled by A-type stochastic integration, thereby effectively transforming the computational representation of the stochastic differential equations into a set of deterministic ordinary differential equations (ODEs) [38,39], ensuring the robustness of the potential landscape under the influence of the noise.

The temporal change in the expression level (i.e., concentration/activity) of a factor is regulated by one or more other factors. We used a Hill function to describe the rate of generation under synergistic action [40,41]. The coarse-grained activation and inhibition follow with

(2) $$f_{activation}=\frac{a{\left[activitor\right]}^{n}}{1+a{\left[activitor\right]}^{n}},$$

(3) $$f_{inhibition}=\frac{1}{1+a{\left[inhibitor\right]}^{n}}.$$

wherein, n and a are the Hill coefficient and the reciprocal of the apparent dissociation constant, respectively. To encompass the behavior of activation and inhibition, the molecular interactions ought to be switch-like, i.e., n and a chosen with $a\approx 1/2^{n}$. Modifying the value would serve as a method for structural perturbation, allowing for the assessment of factors such as network stability. The expression of the factors can all be taken as dimensionless, with the relative levels standardized within the [0–1] range.

### 2.3. Attractors for Cellular States

From the biological perspective, fixed points in the network signify stable patterns of gene expression that remain relatively invariant over time, while they fulfill a particular criterion, denoted as $dx_{i}/dt=0$, in the governing ODEs. These points can be obtained using Newton’s iterative method (cf. the fsolve function in MATLAB), with randomized initial conditions. Stability of the solutions, either stable or transitional, are inferred by the signs of the real part of the eigenvalues of the so-called Jacobian matrix at these fixed points. An all-negative scenario points to equilibrium and, by extension, a stable cellular state. Otherwise, the solution is an unstable saddle point under stochastic perturbation, serving as intermediate states traversed during cell type transitions. Eventually, all stably expressed cell types with sufficiently large basins of attraction are captured when the resulting profiles of steady-state expression remain consistent across multiple orders of simulations (i.e., $10^{5}$, $10^{6}$, $10^{7}$ times).

### 2.4. Validation of Modeling Results

To validate the modeling predictions, the gene expressions of RNAseq data for BC were downloaded from the UCSC Xena database (http://xena.ucsc.edu/, accessed on 19 April 2022), sourced from the TCGA breast cancer (BRCA) database (24 datasets). The expression data contain the value of Log_2_(counts + 1) for each gene. To facilitate the comparison of gene alterations between cancerous and healthy tissues, samples containing normal cellular data were chosen for the relevant representation. Additionally, the Breast Cancer Gene-Expression Miner v4.4 (http://bcgenex.ico.unicancer.fr/BC-GEM/GEM-requete.php?mode=9/, accessed on 4 March 2023), an online dataset featuring statistical mining capabilities, was consulted to consolidate the expression of each factor across various subtypes via DNA microarrays or RNA-seq, serving as an additional reference. All key factors within the network are utilized in the assessment of model-predicted cell types for comparative validation.

### 2.5. Connectivity, Topology, and Phenotypic Landscape

In the vicinity of a saddle point p, minor random vector Δp can be introduced to mimic the shift of cellular states from normal to malignant, taken usually with an amplitude of perturbation in the range $\langle \Delta p\cdot p\rangle \leq 10^{-5}$. These “risk” factors could be induced by internal fluctuations or by external influences such as pharmacological intervention and environmental impact. Under Δp, a transition state would evolve from the initial position along a specific trajectory, ultimately converging to a basin of attraction, representing a stable cellular phenotype. Convergence would be confirmed when the deviation Δx from the stable solution meets $\langle \Delta x\cdot \Delta x\rangle \leq 10^{-5}$. On as many as 1000 repeated realizations, one can readily conclude that the specific saddle point could shift to the deterministic states upon perturbation. Eventually, one can map out all conceivable transition routes between saddle points and stable states, which leads to a phenotypic landscape representing the topology of the endogenous network.

## 3. Results

### 3.1. Construction of Core Endogenous Network for Breast Cancer

**Figure 1:**
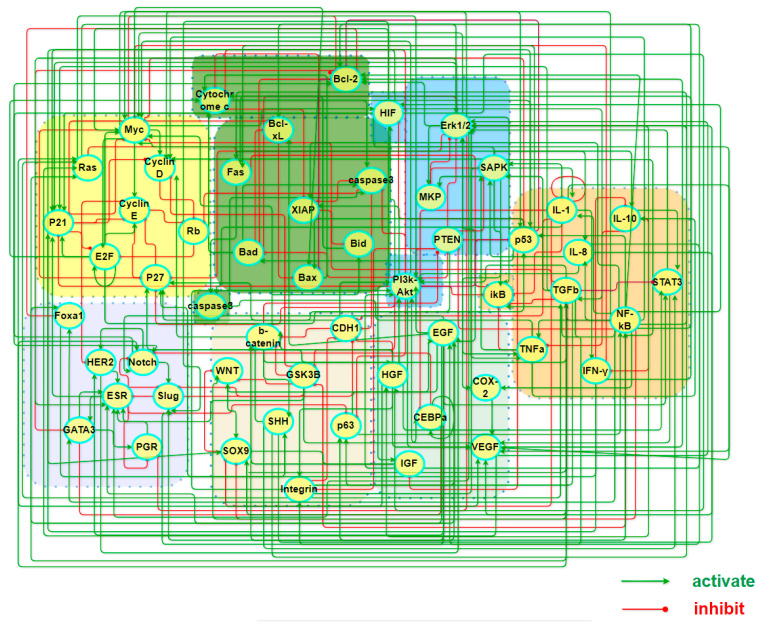
A map of a core endogenous molecular–cellular network for breast cancer. The model includes 7 working modules, 55 factors, and 249 activation/inhibition interactions among them, covering cell cycle, apoptosis, inflammation, metabolism, angiogenesis, cell adhesion, and finally breast cancer specific factors of unique significance.

To comprehend the pathogenic mechanisms and origins of heterogeneous subtypes of BC within stochastic dynamics, we constructed a core endogenous network specifically for the disease, which comprises seven functional modules and collectively incorporates 55 key factors. Table 1 lists the modules along with the key factors underneath each of them, while the interactions between these factors are illustrated in Figure 1. It is worth emphasizing that the selection of these factors was guided by both intrinsic cancer biomarkers and the unique biological features associated with breast cancer.

The network is self-closed with feedback loops of 249 types of signal transduction and transcriptional regulatory relationships. Part A and Table S1 in the Supplementary Materials (SM) has the details of the interactions and the sources of references in the literature. The presence of these loops imparts autonomous decision-making capabilities of the network, enabling it to reach locally steady states with either distinct or ambiguous biological functionalities as it evolves over time. When the basins of attraction for these local stable states are sufficiently large and robust to structural perturbations, they are considered to embody stable cellular states for the tissues. The final expression values in a steady state, with iterative convergence to equilibrium, can be regarded as the relative concentrations or activities of transcription factors or proteins in that particular cellular state. Such identification bridges the gap between molecular mechanisms and phenotypes, making it particularly meaningful to compare these sought states with clinical data or gene expression profiles related to various BC subtypes. Within the system, the network dynamics recovered multiple cell states, including those aligning with the four widely acknowledged intrinsic subtypes of the cancer.

### 3.2. Identification of Cell States and Validation against Clinical Data

Quantitative simulations of network dynamics were carried out via a set of equations in the form of Equation (1). Taking the HER2 factor from Table S1 of SM as an example, which is activated by EGF and Notch factors and inhibited by the CyclinE factor, its relative expression level at time *t*, is ultimately described by a chemical kinetics equation in the form of ODE:

(4) $$\frac{d[HER2]}{dt}=\frac{a\cdot \left({\left[EGF\right]}^{n}+{\left[Notch\right]}^{n}\right)}{1+a\cdot \left({\left[EGF\right]}^{n}+{\left[Notch\right]}^{n}\right)}\times \frac{1}{1+a\cdot \left({\left[CyclinE\right]}^{n}\right)}-[HER2].$$

On the right-hand side of the equation, the first and second factors in the first term indicate, respectively, the effects of activation and inhibition on the production rate of the factor, which can function independently in case of sole activation or inhibition. The second term represents the rate of self degradation. Adopting this structure, the problem converts into analysis of a nonlinear system with 55 dimensions, represented by a series of ODEs. A complete set of the equations can be found in Part B of SM.

**Figure 2:**
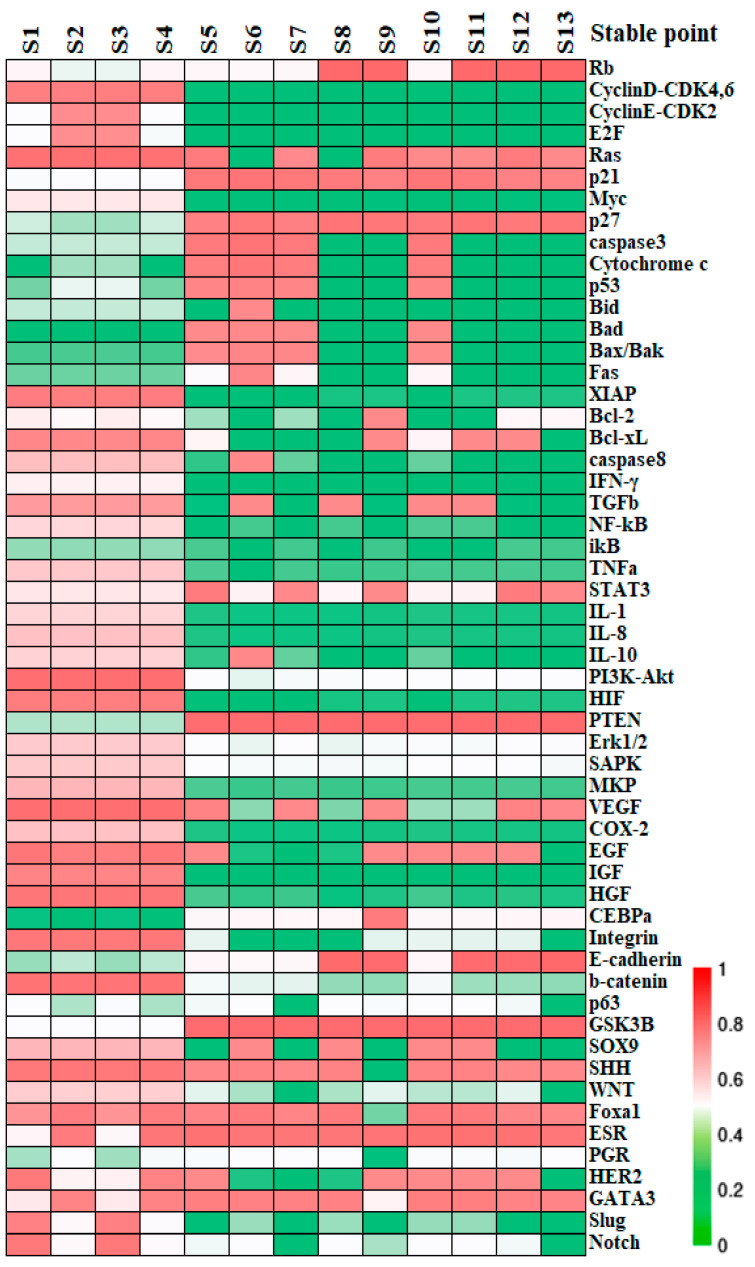
The relative expression of the stable states. The molecular profiles of the stable states obtained from the network dynamics. Each column accounts for a stable state calculated by Newton iteration while the rows list the network factors recruited. The expression levels of factors in each state are shown by colors; “red” represents the maximal expression which corresponds to a value of “1”; “green” denotes minimal expression that its level is not sufficient to affect its target factors significantly.

Stationary point x can be identified upon $dx_{i}/dt=0$ across all equations. If the real part of the eigenvalues of the Jacobian matrix at the point are all negative, the state is asymptotically stable, denoted as a stable state (S). Conversely, a transition state (T) arises when at least one of the eigenvalues has a non-negative real part, serving as a nexus between two or more stable states. Within the 55-dimensional phase space, regardless of whether the values of the parameters n=3,a=8 are rigorously implemented or with structural perturbations, 13 states remain stable, with their profiles illustrated by colors in Figure 2.

Stable states identified by simulations are posited to correlate with specific, or yet-to-be-clarified, biological phenotypes or cell states. Alignment of factors between the activity patterns at the modular level and the established molecular data helps with assessing and predicting the cell destiny. For the functional modules other than the block of BC-specific factors, switch “OFF” or “ON” can be attributed to their operational status. For instance, the apoptosis module is considered “ON” when pro-apoptotic factors Caspase3 and Bad are activated, while anti-apoptotic factors Bcl-2 and Bcl-xL are inactive; otherwise, it is deemed “OFF” [42]. The predicted switch status on all the functional modules is outlined in Table 2. We further assume for simplicity that BC tissue consists primarily of cancer cells, whereas normal breast tissue comprises functionally intact epithelial cells. The 13 stable states are then broadly categorized into cancer states, healthy epithelial states, and apoptotic states. Specifically, states S1–S4 can be recognized as cancer states due to their deactivated apoptosis and adhesion modules, in conjunction with activated inflammation, angiogenesis modules, plus an anaerobic metabolic pathway [37,43,44,45]. And the stable states S8, S9, and S11–S13 exhibit characteristics of quiescent normal breast epithelial cells, with inactive cell cycle and apoptosis modules, no angiogenesis or inflammation, intercellular adhesion, and aerobic metabolism. Other stable states are inferred to be in an apoptotic state due to the active apoptosis module. These functional module statuses in normal breast epithelium and BC were summarized by clinical and experimental data. In other words, the network’s predictions for states S1–S4, or S8, S9, S11–S13 were validated at the modular level and align with the experimental characteristics of both BC cells and the normal breast epithelial cells, as detailed in Table 2. Further information on the factor expression and criteria for the state assessment is provided in Table S2, Part C of SM.

**Figure 3:**
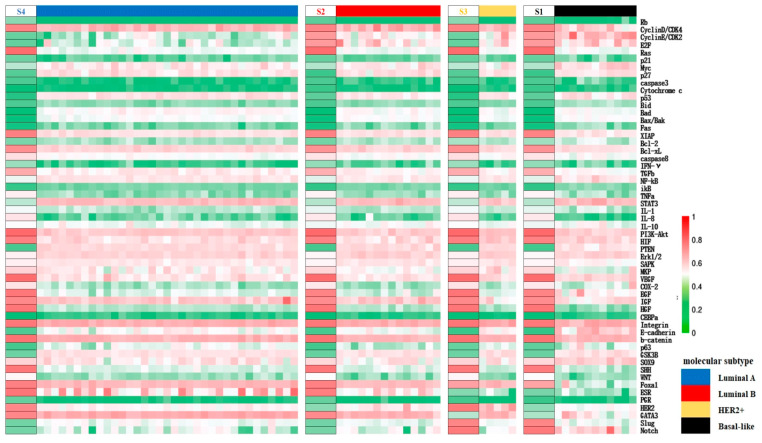
Mapping of the segments of the cancer states obtained by network modeling to the expression data of different molecular subtypes of breast cancer in TCGA upon clustering. The left side of each segment represents the different predicted cancer states listed as S1–S4, and the expression pattern is similar to the expression of clinical data in the corresponding subtype samples from TCGA on the right side columns, indicated in different colors, as shown in the legend. Red to green represent the high to low relative expression levels of the factors, respectively, as in Figure 2.

Distinction within cancerous cell states can be inferred from the expression patterns of BC-specific factors. Specifically, S2 and S4 are identified as Luminal types due to their high ER expression, matching the molecular criteria for the subtype. S1 and S3, with low ER activity, correspond to the HER2+ subtype and basal-like subtype respectively, based on their HER2 and PR expression levels. Cells are in a proliferative state when the cell cycle is in “ON”, or in a quiescent state when the latter is in “OFF”. Consequently, S2 and S4 can be split into Luminal B and Luminal A subtypes based on proliferation rate. These cancer cell types were further validated using high-throughput clinical data: clustering the cancer datasets from the TCGA database, which also contain expression profiles for normal tissue based on the PAM50 classification specified therein, it was shown that the expression patterns of factors in S1–S4 align with the gene expression status of their respective subtypes with a concordance rate exceeding 70%, as seen on the heatmap presented in Figure 3. To summarize, the core endogenous network constructed in this study spontaneously elevates four breast cancer states that correspond to known classifications, thereby confirming the model’s reliability on the molecular scale and its accuracy in predicting the cell types.

### 3.3. Topological Structure and Functional Landscape of Phenotype

**Figure 4:**
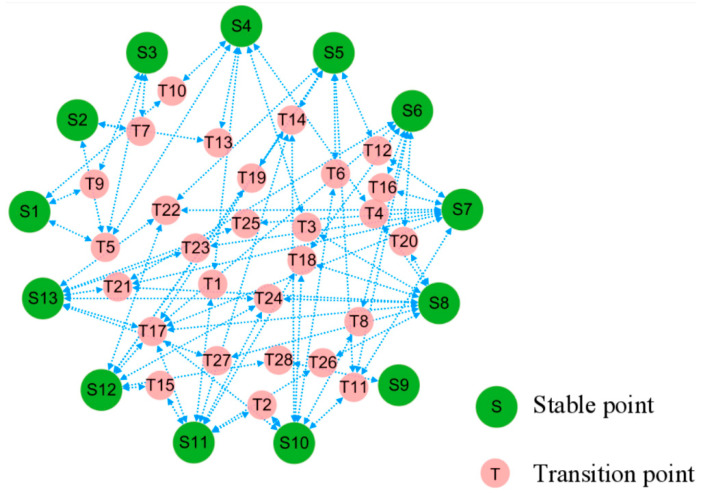
Topological connections among the stable and transitional fixed points. In terms of stochastic dynamics, small-red circles of saddle point sit on higher potential relative to large-green circles of stable point nearby, posing as barriers and pathways between the latter.

Within the stochastic dynamics of positional perturbations, cell evolution pathways are far from random movement. First, cell states tend to transition along paths that minimize the excess stochastic potential. We can connect stable states through the saddle points that reach these equilibrium points after perturbation to obtain a topological diagram, which places constraints on feasible trajectories between cell states. The resulting topology can be regarded as a potential landscape for the stochastic dynamics, cf. Figure 4.

**Figure 5:**
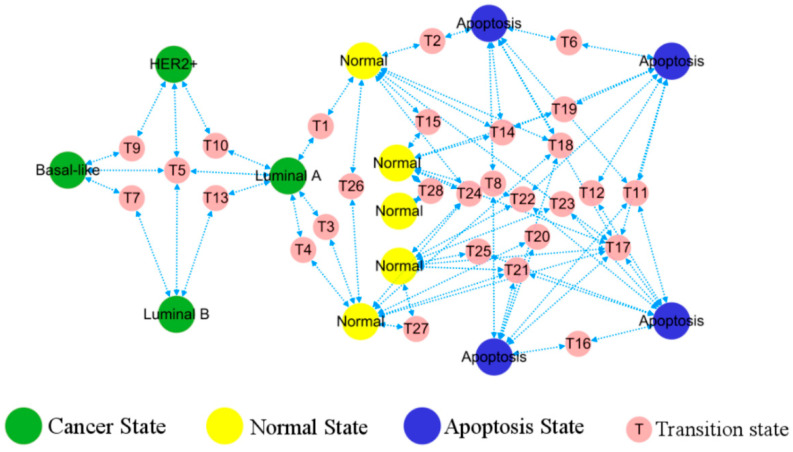
Landscape of breast cancer functional phenotype. Each homeostasis corresponds to a distinct, well-characterized cell phenotype, with their occurrence and transformation in accordance with the depicted routes. Predicted cell types are in a large circle, while the intermediate states bridging their transitions are in a small circle.

The predicted phenotypes correspond to different stable points, consequently the topological diagram becomes a functional phenotypic landscape, providing visual transition paths between various stable states indicative of patterns of cancer development, cf. Figure 5. The simultaneous emergence of different BC, healthy, and apoptotic states within the network lays the ground for tracing of the cell origins on the heterogeneous breast cancer.

### 3.4. Cellular Origins and Heterogeneous Subtypes of Cancer

**Figure 6:**
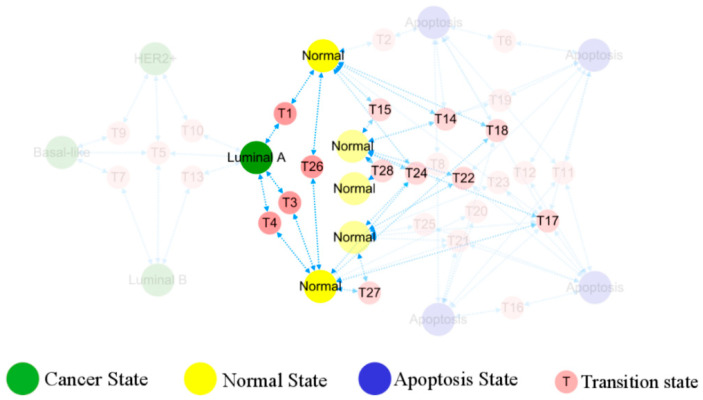
Evolution of normal breast epithelial cells into a cancerous state. The five different normal breast epithelial cells reach the Luminal A subtype of the cancer state through transition states. There is no one-to-one correspondence between normal tissues and the cancer subtypes as the rest, hidden in transparency, do not evolve from TDLU.

**Figure 7:**
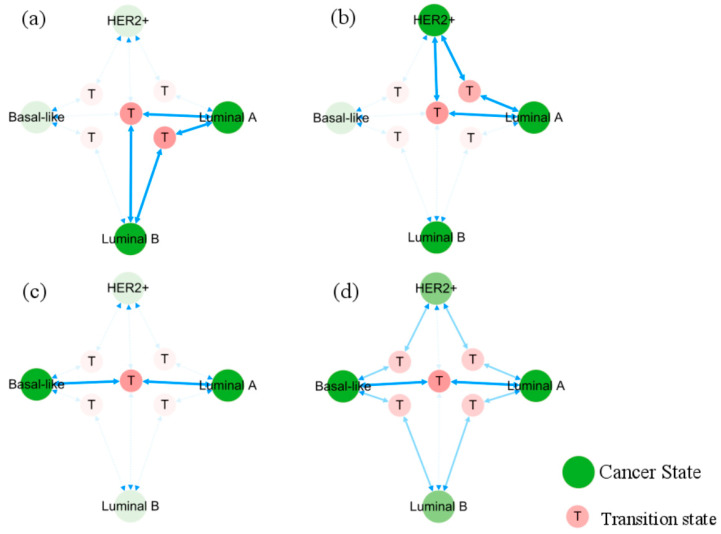
Evolution pathways of heterogeneous breast cancer. Solid blue arrows point to the pathways between phenotypic transitions. Semi-transparent icons/lines indicate multiple step processes. Sub-figures (**a**–**c**) show, respectively, the pathways of Lumina A subtype transforming directly into Luminal B, HER2+, and basal-like subtypes. Sub-figure (**d**) displays all pathways from Luminal A to basal-like subtype.

In Figure 6, the occurrence and transition behaviors of distinct BC subtypes are visually represented in the phenotypic landscape, along with the biological significance of stable states. Different groups of normal cells initially develop into Luminal A BC cells during carcinogenesis. Then Luminal B and HER2+ cancer subtypes can evolve from the Luminal A subtype through two pathways, both can further progress into basal-like breast cancer. However, when Luminal A subtype progresses to basal-like breast cancer, it needs to transition through the Luminal B and HER2+ subtypes, except for the shared T5 transition step with all subtypes. Namely, the process requires more intricate steps, as further depicted in Figure 7.

**Figure 8:**
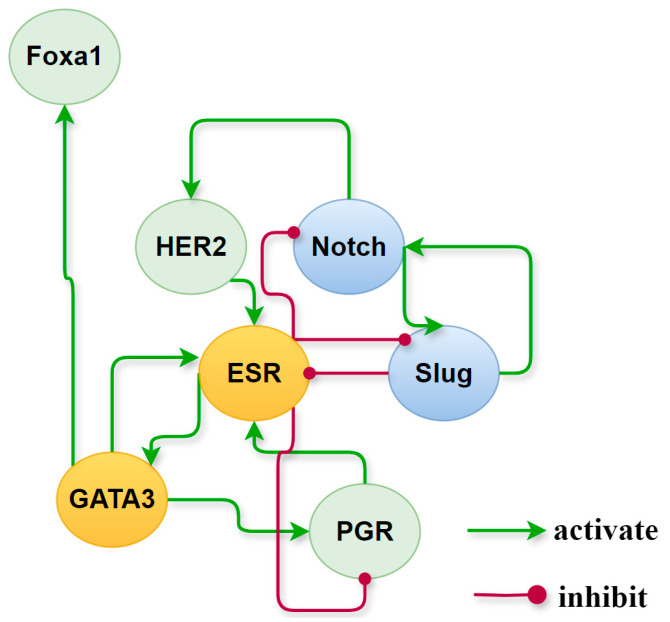
Roles of cancer-specific factors activated in different subtypes of breast cancer. In the yellow circles are the nodes that are continuously activated in the ER+ phenotype which are painted, the blue ones are those that are permanently activated in the ER- medium phenotype, while nodes in the green circles differ in relative expression in different molecular subtypes.

Activated network factors exert significant influence on other factors through their interactions, playing a crucial role in maintaining stable states. Setting 0.5 as the threshold for high relative expression, we have a set of activated cancer-specific factors that forms a subnetwork and maintains the state of each cancer subtype. Their roles and behavior on the subnetwork, extracted from the global endogenous network, are illustrated in Figure 8. A positive feedback loop formed by ER and GATA3 is consistently present in ER+ breast cancer phenotypes, whereas in ER− breast cancer phenotypes, there exists a positive feedback loop on Notch and Slug. To verify the contributions of these two positive feedback loops to the maintenance of BC subtypes and transition between phenotypes, we implemented regulatory adjustments to the relevant dynamic structure within the network dynamics. This involved simulating the blockade and knockout of Slug and ER, which mutually inhibit yet are independently activated. Upon deactivation of ER and Slug, the respective stable states associated with these factors were eliminated. Details on the expression of breast cancer-specific factor modules under the simulated conditions can be found in Table S3, Part D of SM.

## 4. Discussion

This study integrated 55 proteins and transcription factors, along with their regulatory interactions related to the occurrence and development of breast cancer. On this basis, the core endogenous network model of breast cancer was constructed. Different cell fates and various intermediate states arise naturally from the evolutionary dynamics of the network. Similar to how multicellular organisms make use of the same genome to generate and maintain different cell types through nonlinear feedback regulation, the unique network structure formed by the nonlinear biological interactions of endogenous factors cultivates and sustains cancer states of heterogeneous characteristics.

The model takes a coarse-grained approach to network dynamics, employing a set of simple, structured equations to quantify the evolution of cell states. Within a reasonable range of parameters, the three classes of cell states—cancer, normal, and apoptosis all emerge and remain stable, even under structural perturbations. By matching the characteristics of molecular subtypes of BC and comparing the calculated expression with data from the TCGA breast cancer database, we were able to attribute actual biological significance to those cell states identified as in a cancer state. It demonstrates the capability of the model to reproduce both normal breast epithelial and heterogeneous BC states.

The network was constructed with no reference to gene data from breast or other cancers, but rather by, based on molecular biology knowledge, selecting the relevant modules with additional cancer-specific key factors and their regulatory relationships. These selections are designed to reliably and reproducibly describe biological phenomena rather than specifics, thus current focusing on a conservative core network level that has been repeatedly validated. As the spontaneous emergence of cancer states in this unprescribed network correspond to all four widely recognized breast cancer molecular subtypes, it, to some extent, supports the molecular subtype classification standards from the perspective of endogenous network.

Beyond stable cell states, the modeling also predicted the transition states that readily flow towards stable states under local positional perturbations. The connections between perturbed saddle points and stable states suggest feasible routes for cell state transitions. Constructing these pathways into a topological landscape provides a visual representation for the occurrence and transition behaviors of different BC subtypes. Specifically, Luminal A subtype breast cancer, characterized by a lower proliferation rate, acts as the sole bridge between cancer states and normal breast epithelial cell states within the topological landscape. Various normal breast cells can evolve into this subtype through one or multiple steps, which could then further develop into other BC subtypes. Moreover, the cancer states in the landscape cannot transition directly to apoptotic states through transitional states; instead, they must revert to normal epithelial cells before progressing to apoptosis, revealing the root cause of the unlimited growth and incurable nature of cancer cells.

These findings are consistent with the phenomenon that Luminal A breast cancer, which has an incidence rate as high as 70%, has a better prognosis, while the most aggressive basal-like breast cancer has an incidence rate of only about 10%. This also reconciles two seemingly contradictory hypotheses about the origins of breast cancer cells: In our theory, different subtypes of breast cancer do not originate from normal breast epithelial tissues with similar gene expression profiles, nor do they arise from breast stem cells with varying differentiation capabilities or genetic mutations. Instead, the illusion in lineage tracing is caused by the evolutionary progression through a singular subtype of breast cancer.

We further isolated cancer-specific factors activated within different cell states to construct a subnetwork which maintains the characteristics of each subtype. Positive feedback loops formed by ER and GATA3, as well as by Slug and Notch, are essential for maintaining, respectively, the ER+ and ER- phenotypes of breast cancer. Negative feedback, such as the antagonistic interaction between Slug and ER, acts more like a genetic switch that determines phenotype transitions, consistent with findings in the literature [46].

In modeling the core endogenous network, the initial expression of each factor, their interaction relationships, and the dynamic structure parameters can all be qualitatively controlled to simulate the strengthening or weakening of feedback loops that maintain the original network’s stability, when influenced by gene mutations, environmental noise changes, and other factors. Having obtained the key factors and loops that sustain various cell states, further simulations of drug interventions on different factors can provide potential treatments or mitigation strategies for breast cancer. It should be noted that the current network is a coarse-grained core-level construction, omitting many detailed parameters and modules describing more specific functions, allowing for a general summary of the overall evolution of breast cancer phenomena. The incorporation of modules and factors that diverge from the research objectives can complicate the model and obscure critical insights. Furthermore, certain regulatory mechanisms and biochemical parameters may still be inadequately explored. As understanding of breast cancer biology advances and research objectives evolve, expanding the approach by incorporating influences such as the micro-environment effect and cell interactions may gradually bring the network’s evolution closer to realistic biological processes, paving the way for investigating precise and effective breast cancer treatments.

## 5. Conclusions

Aiming to achieve a systemic and quantitative analysis, this work constructed a core endogenous network model of breast cancer using a nonlinear dynamic system. By analyzing the steady states and transitional fixed points, we captured valuable information on the occurrence and evolution of breast cancer. This self-regulating network’s ability to replicate known breast cancer subtypes and predict changes in key factor activities underscores its potential in guiding therapeutic strategies. In fact, some recent progress has been achieved in the search for therapeutic targets of gastric cancer under the endogenous network modeling of clinical data, where an explicit new layer of regulation serves to enhance the study. The approach was able to conduct a “dry experiment” and uncover a combination of multiple targets with a potentially significant reduction in the cancerous basins in silicon [47].

## Figures and Tables

**Table 1:** Modules and factors underneath constituting the core endogenous network.

Module	Factors
Cell cycle	Rb, CyclinD/CDK4, CyclinE/CDK2, E2F, Ras, p21, p27, Myc
Cell apoptosis	caspase3, Cytochrome c, Bid, Bad, Bax, Fas, XIAP, Bcl2, BclxL, caspase8
Inflammation	IFN-γ, TGFb, NF-kB, p53, ikB, TNFa, STAT3, IL-1, IL-8, IL-10
Metabolism	PI3K-Akt, HIF, PTEN, Erk1/2, SAPK, MKP
Angiogenesis	VEGF, COX-2, EGF, IGF, HGF, CEBPa
Adhesion factor	Integrin, E-cadherin, b-catenin, p63, GSK3B, SOX9, SHH, WNT
BC special factor	Foxa1, ER, PR, HER2, GATA3, Slug, Notch

**Table 2:** Switch state of functional modules.

Stable State	Cell Cycle	Cell Apoptosis	Inflammation	Metabolism	Angiogenesis	Adhesion Factor	Cell State
S1	OFF	OFF	ON	ON	ON	OFF	Cancer
S2	ON	OFF	ON	ON	ON	OFF	Cancer
S3	OFF	OFF	ON	ON	ON	OFF	Cancer
S4	ON	OFF	ON	ON	ON	OFF	Cancer
S5	OFF	ON	OFF	OFF	OFF	OFF	Apoptosis
S6	OFF	ON	OFF	OFF	OFF	OFF	Apoptosis
S7	OFF	ON	OFF	OFF	OFF	OFF	Apoptosis
S8	OFF	OFF	OFF	OFF	OFF	ON	Normal
S9	OFF	OFF	OFF	OFF	OFF	ON	Normal
S10	OFF	ON	OFF	OFF	OFF	OFF	Apoptosis
S11	OFF	OFF	OFF	OFF	OFF	ON	Normal
S12	OFF	OFF	OFF	OFF	OFF	ON	Normal
S13	OFF	OFF	OFF	OFF	OFF	ON	Normal

## Data Availability

The data that support the findings of this study are available from the corresponding author upon reasonable request.

## Supplementary Materials

The following supporting information can be downloaded at: https://www.mdpi.com/article/10.3390/biology13080564/s1, Table S1: Abbreviation of genes/molecules and their mutual interactions; Table S2: Expression of module factors and switch vs. steady states; Table S3: Expression of BC specific factors under simulations; List S1: Literature sources cited in Table S1; List S2: ODEs for the endogenous network.
