# Supplementary material for: Heterogeneous Evolution of Breast Cancer Cells—An Endogenous Molecular-Cellular Network Study
Source: Biology (Basel). 2024 Jul 26;13(8):564. doi: 10.3390/biology13080564 (PMC11352015; doi:10.3390/biology13080564)
Supplement: Supplementary file 1 [file biology-13-00564-s001.zip › biology-3111075-supplementary.pdf]

Supplementary Materials

Heterogeneous Evolution of Breast Cancer Cells—An Endogenous Molecular-Cellular Network Study

Tianqi Li [1], Yong-Cong Chen [1,*] and Ping Ao [2]

[1] Center for Quantitative Life Sciences & Physics Department, Shanghai University, Shanghai 200444, China
[2] School of Biomedical Engineering, Sichuan University, Chengdu 610065, China
* Correspondence: chenyongcong@shu.edu.cn

This file contains the supplementary materials referred in the main article entitled above. It includes details of the bio-process simulations, the computational model and the parameters involved in the study. They consist of tables of gene / molecular factors along with their mutual interactions, a list of literature sources, and a complete set of differential equations used in the analysis. Some results of tentative inhibiting and knock-out alternations are presented at the end of the file.

A. Genes and/or molecules (factors) that are selected in the endogenous network modeling. The literature sources for the interactions on each of the network factors are listed after the table.

Table S1. Abbreviation of genes/molecules and their mutual interactions

**Factors**	Actived by	Inhibited by	**Factors**	Actived by	Inhibited by
X1=Rb		X2=Cyclin D-CDK4,6[1]	X29=PI3K-Akt	X39=HGF[111]	
		X3=Cyclin E-CDK2[2]		X35=VEGF[112]	
		X9=caspase3[3]		X42=E-cadherin [113]	
X2=Cyclin D-CDK4,6	X8=Myc[4][5]	X7=p21[10]		X41=Integrin[19]	
	X43=b-catenin[6]	X45=GSK3B[11]		X37=EGF[102]	
	X21=NF-kB[7]			X38=IGF[114]	
	X32=Erk1/2[8]			X5=Ras[115]	X31=PTEN[116]
	X33=SAPK[9]			X20=TGFb[117]	
X3=Cyclin E-CDK2	X4=E2F[12]	X7=p21[10]		X27=IL-8[118]	
	X8=Myc[12]	X6=p27[13]	X30=HIF	X55=Notch[119]	X22=p53[120]
X4=E2F	X8=Myc[14]	X1=Rb[2]		X8=Myc[121]	
	X4=E2F[15]	X7=p21[16]		X29=PI3K-Akt [122]	
X5=Ras	X37=EGF[17]		X31=PTEN		X21=NF-kB[123]
	X35=VEGF[18]				X29=PI3K-Akt[124]
	X41=Integrin[19]				X43=b-catenin [125]
	X39=HGF[20]		X32=Erk1/2	X5=Ras[126]	X34=MKP[91]
X6=p27	X31=PTEN[21]	X32=Erk1/2[25]		X41=Integrin [127]	X31=PTEN[128]
	X42=E-cadherin [22]	X8=Myc[26]		X47=SHH[28]	
	X19=IFN-γ[23]	X29=PI3K-Akt[27]		X25=STAT3[85]	
	X20=TGFb[24]	X3=Cyclin E-CDK2[28]	X33=SAPK	X20=TGFb[129]	X31=PTEN [132]
X7=p21	X22=p53[29]	X29=PI3K-Akt[34]		X24=TNFa[130]	X34=MKP[133]
	X4=E2F[30]	X8=Myc[35]		X26=IL-1[131]	
	X20=TGFb[31]			X27=IL-8[118]	
	X30=HIF[32]			X37=EGF[17]	
	X19=IFN-γ[23]			X35=VEGF[134]	
	X32=Erk1/2[30]			X47=SHH[92]	
	X50=ESR[33]		X34=MKP	X32=Erk1/2[91]	
X8=Myc	X32=Erk1/2[36]	X20=TGFb[35]		X33=SAPK[135]	
	X43=b-catenin[37]	X19=IFN-γ[41]	X35=VEGF	X30=HIF[136]	
	X33=SAPK[38]	X7=p21[30]		X25=STAT3[137]	
	X21=NF-kB[49]	X22=p53[42]		X27=IL-8[118]	
	X30=HIF[40]			X39=HGF[40]	
	X4=E2F[36]			X36=COX-2[138]	
	X29=PI3K-Akt [38]			X26=IL-1[139]	
X9=caspase3	X18= caspase8,10[43]	X15=XIAP[46]		X41=Integrin [19]	
	X10= Cytochrome c[44]			X29=PI3K-Akt [140]	
	X12=Bad[45]		X36=COX-2	X21=NF-kB[62]	
X10= Cytochrome c	X12=Bad[47]	X29=PI3K-Akt [50]		X33=SAPK[141]	
	X13=Bax[48]	X15=XIAP[51]	X37=EGF	X27=IL-8[118]	
	X11=Bid[47]			X41=Integrin[19]	
	X4=E2F[49]			X44=p63[142]	

**Factors**	Actived by	Inhibited by	**Factors**	Actived by	Inhibited by
X11=Bid	X17=caspase8,10 [52]	X15=XIAP[51]		X52=HER2[143]	
X12=Bad	X22=p53[53]	X32=Erk1/2[54]	X38=IGF	X30=HIF[91]	X22=p53[144]
		X29=PI3K-Akt [55]	X39=HGF	X32=Erk1/2[145]	
X13=Bax	X8=Myc[56]	X16=Bcl-2[58]		X30=HIF[146]	
	X22=p53[57]	X17=Bcl-xL[59]		X43=b-catenin [147]	
X14=Fas	X22=p53[60]	X5=Ras[63]	X40=CEBPa	X40=CEBPa[148]	X26=IL-1[150]
	X21=NF-kB[62]			X45=GSK3B[149]	X53=GATA3[151]
			X41=Integrin	X35=EGF[152]	X31=PTEN[127]
	X19=IFN-γ[63]				
X15=XIAP	X21=NF-kB [61][64]	X10= Cytochrome c[66]		X39=HGF[152]	X26=IL-1[153]
	X29=PI3K-Akt [65]		X42= E-cadherin		X41=Integrin [113]
X16=Bcl-2	X32=Erk1/2[67]	X8=Myc[56]			X39=HGF[103]
	X55=Notch[68]	X12=Bad[71]			X9=caspase3 [154]
	X21=NF-kB[69]	X22=p53[72]			X54=Slug[155]
	X35=VEGF[70]	X49=Foxa1[73]	X43= b-catenin	X47=SHH[156]	X42=E-cadherin [160]
X17=Bcl-xL	X37=EGF[69]	X12=Bad[47]		X39=HGF[157]	X45=GSK3B [161]
	X38=IGF[74]	X8=Myc[56]		X37=EGF[158]	
	X21=NF-kB[75]			X38=IGF[159]	
X18= caspase8,10	X14=Fas[47]		X44=p63	X20=TGFb[162]	X50=ESR[164]
	X24=TNFa[76]			X37=EGF[163]	X29=PI3K-Akt [165]
	X26=IL-1[77]		X45= GSK3B		X29=PI3K-Akt [166]
X19=IFN-γ	X26=IL-1[78]	X28=IL-10[79]			X21=NF-kB [167]
X20=TGFb	X41=Integrin[80]	X21=NF-kB[62]	X46=SOX9	X30=HIF[168]	X26=IL-1[171]
	X30=HIF[81]			X20=TGFb[169]	X21=NF-kB[171]
	X46=SOX9[82]			X48=WNT[170]	
X21=NF-kB	X33=SAPK[83]	X23=ikB[62]	X47=SHH	X43=b-catenin [156]	
	X44=p63[84]	X25=STAT3[85]		X49=Foxa1[172]	
X22=p53	X8=Myc[36]	X29=PI3K-Akt[89]	X48=WNT	X37=EGF[173]	X45=GSK3B [173]
	X4=E2F[86]			X20=TGFb[174]	X46=SOX9[175]
	X21=NF-kB[87]		X49=Foxa1	X20=TGFb[176]	
	X10= Cytochrome c[88]			X53=GATA3 [177][178]	
X23=ikB	X21=NF-kB[90]	X24=TNFa[93]	X50=ESR	X37=EGF[179]	X54=Slug[185]
	X32=Erk1/2[91]	X26=IL-1[93]		X45=GSK3B [180]	
	X33=SAPK[92]	X29=PI3K-Akt[94]		X51=PGR[181]	
		X14=Fas[95]		X53=GATA3[182]	
		X20=TGFb[96]		X52=HER2[183]	
X24=TNFa	X33=SAPK[97]	X28=IL-10[99]		X32=Erk1/2[184]	
	X32=Erk1/2[97]		X51=PGR	X53=GATA3[186]	X50=ESR[187]
	X21=NF-kB[98]				X32=Erk1/2[188]
X25=STAT3	X28=IL-10[100]	X20=TGFb[104]	X52=HER2	X37=EGF[189]	X3=Cyclin E-CDK2[191]
	X47=SHH[101]			X55=Notch[190]	
	X37=EGF[102]		X53=GATA3	X21=NF-kB[192]	X16=Bcl-2
	X39=HGF[103]			X50=ESR[193]	

Factors	Actived by	Inhibited by	Factors	Actived by	Inhibited by
X26=IL-1	X33=SAPK[105]<br>X21=NF-kB[106]	X26=IL-1[107]	X54=Slug	X30=HIF[194]<br>X21=NF-kB[195]<br>X20=TGFb[196]<br>X55=Notch[197]	X45=GSK3B [198][199]<br>X50=ESR[200]
X27=IL-8	X21=NF-kB[108]<br>X33=SAPK[105]				
X28=IL-10	X24=TNFa[100]<br>X14=Fas[109]	X19=IFN-γ[110]	X55=Notch	X30=HIF [201][202]<br>X20=TGFb[203]<br>X37=EGF[204]	X40=CEBPa [205]<br>X50=ESR[206]

List S1. Litereature sources cited in Table S1

[1] Peurala E, Koivunen P, Haapasaari, K M, et al. The prognostic significance and value of cyclin D1, CDK4 and p16 in human breast cancer. Breast Cancer Research, 2013, 15(1): R5.

[2] Robert R J. Cyclin-dependent protein serine/threonine kinase inhibitors as anticancer drugs. Pharmacological Research. 2019, 139: 471-488.

[3] Katsuda K, Kataoka M, Uno F, et al. Activation of caspase-3 and cleavage of rb are associated with P16-mediated apoptosis in human non-small cell lung cancer cells. Oncogene, 2002, 21(13): 2108-2113.

[4] Obaya A J, Mateyak M K, Sedivy J M. Mysterious liaisons: the relationship between c-Myc and the cell cycle[J]. Oncogene, 1999, 18(19): 2934-2941.

[5] Wang C, Lisanti M P, Liao D J. Reviewing once more the c-myc and Ras collaboration: converging at the cyclin D1-CDK4 complex and challenging basic concepts of cancer biology. Cell Cycle, 2011, 10(1): 57-67.

[6] Tetsu O, McCormick F. β-Catenin regulates expression of cyclin D1 in colonc carcinoma cells. Nature, 1999, 398(6726): 422-426.

[7] Guttridge D C, Albanese C, Reuther J Y, et al. NF-kappaB controls cell growth and differentiation through transcriptional regulation of cyclin D1. Molecular Cell Biology, 1999, 19(8): 5785-99.

[8] Chang F, Steelman L S, Lee J T, et al. Signal transduction mediated by the Ras/Raf/MEK/ERK pathway from cytokine receptors to transcription factors: potential targeting for therapeutic intervention. Leukemia, 2003, 17(7): 1263-1293.

[9] Ding J, Ning B F, Huang Y, et al. PI3K/Akt/JNK/c-Jun signaling pathway is a mediator for arsenite-induced cyclin D1 expression and cell growth in human bronchial epithelial cells. Current Cancer Drug Targets, 2009, 9(4): 500-509.

[10] Sherr C J, Roberts J M. CDK inhibitors: positive and negative regulators of G1-phase progression. Genes & Development, 1999,13(12): 1501-1512.

[11] Lee Y, Dominy J E, Choi Y J, et al. Cyclin D1-Cdk4 controls glucose metabolism independently of cell cycle progression. Nature, 2014, 510(7506): 547-551.

[12] Leone G, DeGregori J, Sears R, et al. Myc and Ras collaborate in inducing accumulation of active Cyclin E/Cdk2 and E2F. Nature, 1997, 387(6631): 422-426.

[13] Ilyin G P, Glaise D, Gilot D, et al. Regulation and role of p21 and p27 cyclin dependent kinase inhibitors during hepatocyte differentiation and growth. American Journal of Physiology-Gastrointestinal and Liver Physiology, 2003, 285(1): 115-127.

[14] Weinberg R A. E2F and cell proliferation: A world turned upside down. Cell, 1996, 85(4): 457-459.

[15] Engelmann D, Putzer B M. The dark side of E2F1: in transit beyond apoptosis. Cancer Research, 2012, 72 (3): 571-575.

[16] Afshari C A, Nichols M A, Xiong Y, et al. A role for a p21-E2F interaction during senescence arrest of normal human fibroblasts. Cell Growth Differ, 1996, 7(8): 979-988.

[17] Citri A, Yarden Y. EGF–ERBB signalling: Towards the systems level. Nature Reviews Molecular Cell Biology, 2006, 7(7): 505-516.

[18] Serban D, Leng J, Cheresh D. H-ras regulates angiogenesis and vascular permeability by activation of distinct downstream effectors. Circulation Research, 2008, 102(11): 1350-1358.

[19] Giancotti F G, Ruoslahti E. Integrin signaling. Science, 1999, 285(5430): 1028-1033.

[20] Gherardi E, Birchmeier W, Birchmeier C, et al. Targeting MET in cancer: Rationale and progress. Nature Reviews Cancer, 2012, 12(2): 89-103.

[21] Gottschalk A R, Basila D, Wong M, et al. p27Kip1 is required for PTEN-induced G1 growth arrest. Cancer Research, 2001, 61(5):

2105-2111.
[22] St. Croix B, Sheehan C, Rak J W, et al. E-cadherin– Dependent growth suppression is mediated by the cyclin-dependent kinase inhibitor p27KIP1. The Journal Of Cell Biology, 1998, 142(2): 557-571.
[23] Mandal M, Bandyopadhyay D, Goepfert T M, et al. Interferon-induces expression of cyclin-dependent kinase-inhibitors p21 WAF1 and p27 Kip1 that prevent activation of cyclin-dependent kinase by CDK-activating kinase (CAK). Oncogene, 1998, 16(2): 217-225.
[24] Lecanda J, Parekh T V, Gama P, et al. Transforming growth factor-beta, estrogen, and progesterone converge on the regulation of p27Kip1 in the normal and malignant endometrium. Cancer Research, 2007, 67(3): 1007-1018.
[25] Ishida N, Kitagawa M, Hatakeyama S, et al. Phosphorylation at serine 10, a major phosphorylation site of p27(Kip1), increases its protein stability. Journal of Biology Chemistry, 2000, 275(33): 25146-25154.
[26] Yang W, Shen J, Wu M, et al. Repression of transcription of the p27(Kip1) cyclin-dependent kinase inhibitor gene by c-Myc. Oncogene, 2001, 20(14): 1688-1702.
[27] Sa G, Stacey D W. p27 expression is regulated by separate signaling pathways, downstream of Ras, in Each cell cycle phase. Experimental Cell Research, 2004, 300(2): 427-439.
[28] Sheaff R J, Groudine M, Gordon M, et al. Cyclin E-CDK2 is a regulator of p27Kip1. Genes & Development, 1997, 11(11): 1464-1478.
[29] Brugarolas J, Chandrasekaran C, Gordon J I, et al. Radiation-induced cell cycle arrest compromised by p21 deficiency. Nature, 1995, 377(6549): 552-557.
[30] Abbas T, Dutta A. p21 in cancer: Intricate networks and nultiple activities. Nature Reviews Cancer, 2009, 9(6): 400-414.
[31] Massagué J. TGFβ in cancer . Cell, 2008, 134(2): 215-230.
[32] Koshiji M, Kageyama Y, Pete E A, et al. HIF-1α induces cell cycle arrest by functionally counteracting Myc. The EMBO Journal, 2004, 23(9): 1949-1956.
[33] Lazennec G, Bresson D, Lucas A, et al. ER beta inhibits proliferation and invasion of breast cancer cells. Endocrinology, 2001, 142(9):4120-4130.
[34] Héron-Milhavet L, Franckhauser C, Rana V, et al. Only Akt1 is required for proliferation, while Akt2 promotes cell cycle exit through p21 binding. Molecular and Cellular Biology, 2006, 26(22):8267-8280.
[35] Riggelen J V, Müller J, Otto T, et al. The interaction between Myc and Miz1 is required to antagonize TGFbeta-dependent autocrine signaling during lymphoma formation and maintenance. Genes & Development, 2010, 24(12):1281-1294.
[36] Dang C V. MYC on the path to cancer. Cell, 2012, 149(1): 22-35.
[37] Sierra J, Yoshida T, Joazeriro C A, et al. The APC tumor suppressor counteracts beta-catenin activation and H3K4 methylation at Wnt target genes. Genes & Development, 2006, 20(5):586-600.
[38] Zhu J, Blenis J, Yuan J. Activation of PI3K/Akt and MAPK pathways regulates Myc mediated transcription by phosphorylating and promoting the degradation of Mad1[C]. Proceedings of the National Academy of Sciences, 2008, 105(18): 6584-6589.
[39] Hinz M, Krappmann D, Eichten A, et al. NF-κB function in growth control: Regulation of Cyclin D1 expression and G0/G1-to-S-phase transition. Molecular and Cellular Biology, 1999, 19(4): 2690-2698.
[40] Zhang Y W, Su Y, Volpert O V, et al. Hepatocyte growth factor/scatter factor mediates angiogenesis through positive VEGF and negative thrombospondin 1 regulation. Proceedings of the National Academy of Sciences, 2003, 100(22): 12718.
[41] Vairo G, Vadiveloo P K, Royston A K, et al. Deregulated c-myc expression overrides IFN gamma-induced macrophage growth arrest. Oncogene, 1995, 10(10): 1969-1976.
[42] Ho J S, Ma W, Mao D Y, et al. p53-Dependent transcriptional repression of c-myc is required for G1 cell cycle arrest. Molecular and Cellular Biology, 2005, 25(17): 7423-7531.
[43] Walczak H, Kantari C. Caspase-8 and bid: caught in the act between death receptors and mitochondria. Biochimica et Biophysica Acta, 2011, 813(4): 558-563.
[44] Adrain C, Martin S J. The mitochondrial apoptosome: A killer unleashed by the cytochrome seas. Trends in Biochemical Science, 2001, 26(6): 390-397.
[45] Chiu C L, Wu J L, Her G M, et al. Aquatic birnavirus capsid protein, VP3, induces apoptosis via the Bad-mediated mitochondria pathway in fish and mouse cells. Apoptosis, 2010, 15(6): 653-668.
[46] Suzuki Y, Imai Y, Nakayama H, et al. A serine protease, HtrA2, is released from the mitochondria and interacts with XIAP, inducing cell death. Molecular Cell, 2011, 8(3): 613-621.
[47] Igney F H, Krammer P H. Death and anti-death: tumour resistance to apoptosis. Nature Reviews Cancer, 2002, 2(4): 277-288.

[48] Youle R J, Strasser A. The BCL-2 protein family: opposing activities that mediate cell death. Nature Reviews Molecular Cell Biology, 2008, 9(1): 47-59.
[49] Moroni M C, Hickman E S, Denchi E L, et al. Apaf-1 is a transcriptional target for E2F and p53. Nature Cell Biology, 2001, 3(6): 552-558.
[50] Fujita E, Jinbo A, Matuzaki H, et al. Akt phosphorylation site found in human caspase-9 is absent in mouse caspase-9. Biochemical and Biophysical Research Communications, 1999, 264(2): 550-555.
[51] Deveraux Q L, Reed J C. IAP family proteins- suppressors of apoptosis. Genes & Development, 1999, 13(3): 239-252.
[52] Li H, Zhu H, Xu C J, et al. Cleavage of BID by caspase 8 mediates the mitochondrial damage in the Fas pathway of apoptosis. Cell, 1998, 94(4): 491-501.
[53] Jiang P, Du W, Wu M. p53 and Bad: Remote strangers become close friends. Cell Research, 2007, 17(4): 283-285.
[54] Zha J, Harada H, Yang E, et al. Serine phosphorylation of death agonist BAD in response to survival factor results in binding to 14-3-3 not BCL-X(L). Cell, 1996, 87(4): 619-628.
[55] Rönnstrand L. Signal transduction via the stem cell factor receptor/c-Kit. Cellular and Molecular Life Sciences, 2004, 61(19-20): 2535-2548.
[56] Hoffman B, Liebermann D A. Apoptotic signaling by c-MYC. Oncogene, 2008, 27(50): 6462-6472.
[57] Miyashita T, Reed J C. Tumor suppressor p53 is a direct transcriptional activator of the human bax gene. Cell, 1995, 80(2):293-299.
[58] Yin X M, Oltvai Z N, Korsmeyer S J. BH1 and BH2 domains of Bcl-2 are required for inhibition of apoptosis and heterodimerization with Bax. Nature, 1994, 369(6478):321-323.
[59] Chipuk J E, Bhat M, Hsing A Y, et al. Bcl-xL blocks transforming growth factor-β1-induced apoptosis by inhibiting Cytochrome c release and not by directly antagonizing Apaf-1-dependent caspase activation in prostate epithelial cells. Journal of Biological Chemistry, 2001, 276(28): 26614-26621.
[60] Müllerp M, Wilder S, Bannasch D, et al. p53 activates the CD95 (APO-1/Fas) gene in response to DNA damage by anticancer drugs. Journal of Experimental Medicine, 1998, 188(11): 2033–2045.
[61] Pahl H L. Activators and target genes of Rel/NF-κB transcription factors. Oncogene, 1999, 18(49): 6853-6866.
[62] Xu X, Fu X Y, Plate J, et al. IFN-γ induces cell growth inhibition by Fas-mediated apoptosis: Requirement of STAT1 protein for up-regulation of Fas and FasL expression. Cancer Research, 1998, 58(13): 2832-2837.
[63] Peli J, Schröter M, Rudaz C, et al. Oncogenic Ras inhibits Fas ligand-mediated apoptosis by downregulating the expression of Fas. The EMBO Journal, 1999, 18(7): 1824-1831.
[64] Karin M, Lin A. NF-kappaB at the crossroads of life and death. Nature Immunology, 2002, 3(3): 221-227.
[65] Dan H C, Sun M, Kaneko S, et al. Akt phosphorylation and stabilization of X-linked inhibitor of apoptosis protein (XIAP). Journal of Biological Chemistry, 2004, 279(7): 5405-5412.
[66] Kale J, Liu Q, Leber B, et al. Shedding Light on Apoptosis at Subcellular Membranes. Cell, 2012, 151(6): 1179-1184.
[67] Catz S D, Johnson J L. Transcriptional regulation of bcl-2 by nuclear factor κB and its significance in prostate cancer. Oncogene, 2001, 20(50): 7342-7351.
[68] Weerkamp F, Luis T C, Naber B A E, et al. Identification of Notch target genes in uncommitted T-cell progenitors: No direct induction of a T-cell specific gene program. Leukemia, 2006, 20(11): 1967-1977.
[69] Breitschopf K, Haendeler J, Malchow P, et al. Posttranslational modification of Bcl-2 facilitates its proteasome-dependent degradation: molecular characterization of the involved signaling pathway. Molecular and Cellular Biology, 2000, 20(5): 1886-1896.
[70] Bufalo D D, Trisciuoglio D, Milella M. Crosstalk between VEGF and Bcl-2 in tumor progression and angiogenesis. Madame Curie Bioscience Database[OL], 2013.
[71] Chen L, Willis S N, Wei A, et al. Differential targeting of prosurvival Bcl-2 proteins by their BH3-only ligands allows complementary apoptotic function. Molecular Cell, 2005, 17(3): 393-403.
[72] Vaseva A V, Moll U M. The mitochondrial p53 pathway. Biochimica et Biophysica Acta, 2009, 1787(5): 414-20.
[73] Song L, Wei X, Zhang B, et al. Role of Foxa1 in regulation of bcl2 expression during oxidative-stress-induced apoptosis in A549 type II pneumocytes. Cell Stress and Chaperones, 2009, 14(4): 417-425.
[74] Sekharam M, Zhao H, Sun M, et al. Insulin-like growth factor 1 receptor enhances invasion and induces resistance to apoptosis of colon cancer cells through the Akt/Bcl-xL pathway. Cancer Research, 2003, 63(22): 7708-7716.
[75] Karin M, Lin A. NF-kappaB at the crossroads of life and death. Nature Immunology, 2002, 3(3): 221-227.

[76] Salvesen G S, Riedl S J. Caspase mechanisms. Programmed Cell Death in Cancer Progression and Therapy, 2008, 615: 13-23.
[77] Janssens S, Beyaert R. A universal role for MyD88 in TLR/IL-1R-mediated signaling. Trends in Biochemical Sciences, 2002, 27(9): 474-482.
[78] Golab J, Zagozdzon, Stoklosal T, et al. Direct stimulation of macrophages by IL-12 and IL-18-a bridge too far?. Immunology Letters, 2000, 72(3): 153-157.
[79] Cope A, Le Friec G, Cardone J, et al. The Th1 life cycle: Molecular control of IFN-γ to IL-10 switching. Trends in Immunology, 2011, 32(6): 278-286.
[80] Munger J S, Huang X, Kawakatsu H, et al. The integrin alpha v beta 6 binds and activates latent TGF beta 1: A mechanism for regulating pulmonary inflammation and fibrosis. Cell, 1999, 96(3): 319-328.
[81] Gaber T, Dziurla R, Tripmacher R, et al. Hypoxia inducible factor (HIF) in rheumatology: low O2! See what HIF can do!. Annals of the Rheumatic Diseases, 2005, 64(7): 971-980.
[82] Ma Q, Ma Y, Dai X, et al. Regeneration of functional alveoli by adult human SOX9+ airway basal cell transplantation. Protein & Cell, 2018, 9(3): 267-282.
[83] Finco T S, Westwick J K, Norris J L, et al. Oncogenic Ha-Ras-induced signaling activates NF-kappaB transcriptional activity, which is required for cellular transformation. Journal of Biological Chemistry, 1997, 272(39): 24113-24116.
[84] King K E, George A L, Sakakibara N, et al. Intersection of the p63 and NF-κB pathways in epithelial homeostasis and disease. Molecular Carcinogenesis, 2019, 58(9): 1571-1580.
[85] Murray P J. Understanding and exploiting the endogenous Interleukin-10/STAT3- mediated anti-inflammatory response. Current Opinion in Pharmacology, 2006, 6(4): 379-386.
[86] Sherr C J. The INK4a/ARF network in tumour suppression. Nature Reviews Molecular Cell Biology, 2001, 2(10): 731-737.
[87] Aleyasin H, Cregan S P, Iyirhiaro G, et al. Nuclear factor-(kappa)B modulates the p53 response in neurons exposed to DNA damage. The Journal of Neuroscience, 2004, 24(12): 2963-2973.
[88] Cregan S P, Dawson V L, Slack R S. Role of AIF in Caspase-dependent and Caspase independent cell death. Oncogene, 2004, 23(16): 2785-2796.
[89] Frisch S M, Francis H. Disruption of epithelial cell-matrix interactions induces apoptosis. Journal of Cell Biology, 1994, 124(4): 619-626.
[90] Sun S C, Ganchi P A, Ballard D W, et al. NF-kappa B controls expression of inhibitor I kappa B alpha: Evidence for an inducible autoregulatory pathway. Science, 1993, 259(5103): 1912-1915.
[91] Madhala-Levy D, Williams V C, Hughes S M, et al. Cooperation between Shh and IGF-I in promoting myogenic proliferation and differentiation via the MAPK/ERK and PI3K/Akt pathways requires Smo activity. Journal of Cellular Physiology, 2012, 227(4): 1455-1464.
[92] Zhu S, Ye Y, Shi Y, et al. Sonic hedgehog regulates proliferation, migration and invasion of synoviocytes in rheumatoid arthritis via Jnk signaling. Frontiers in Immunology, 2020, 11: 1300.
[93] Zandi E, Karin M. Bridging the gap: Composition, regulation, and physiological function of the IkappaB kinase complex. Molecular and Cellular Biology, 1999, 19(7): 4547-4551.
[94] Romashkova J A, Makarov S S. NF-kappaB is a target of AKT in anti-apoptotic PDGF signalling. Nature, 1999, 401(6748): 86-90.
[95] Schneider P, Thome M, Burns K, et al. TRAIL receptors 1 (DR4) and 2 (DR5) signal FADD-dependent apoptosis and activate NF-kappaB. Immunity, 1997, 7(6): 831-836.
[96] Derynck R, Zhang Y E. Smad-dependent and Smad-independent pathways in TGF-β family signalling. Nature, 2003, 425(6958): 577-584.
[97] Rutault K, Hazzalin C A, Mahadevan L C. Combinations of ERK and p38 MAPK inhibitors ablate tumor necrosis factor-α (TNF-α) mRNA induction: Evidence for selective destabilization of TNF-α transcripts. Journal of Biological Chemistry, 2001, 276(9): 6666-6674.
[98] Tidball J G, Villalta S A. Regulatory interactions between muscle and the immune system during muscle regeneration. Regulatory, Integrative and Comparative Physiology, 2010, 298(5): R1173-1187.
[99] Rajasingh J, Bord E, Luedemann C, et al. IL-10-induced TNF-alpha mRNA destabilization is mediated via IL-10 suppression of p38 MAP kinase activation and inhibition of HuR expression. Federation of American Societies for Experimental Biology, 2006, 20(12): 2112-2114.
[100] Saraiva M, O'garra A. The regulation of IL-10 production by immune cells. Nature Reviews immunology, 2010, 10(3): 170-181.

[101] Patel S S, Acharya A, Ray R S, et al. Cellular and molecular mchanisms of curcumin in prevention and treatment of disease. Critical Reviews in Food Science and Nutrition, 2020, 60(6): 887-939.
[102] Marmor M D, Skaria K B, Yarden Y. Signal transduction and oncogenesis by ErbB/HER receptors. International Journal of Radiation Oncology Biology Physics, 2003, 58(3): 903-913.
[103] Shen Y Y, Chen Q W, Li L H. Endostar regulates EMT, migration and invasion of lung cancer cells through the HGF-Met pathway. Molecular and Cellular Probes, 2019, 45: 57-64.
[104] Starsichova A, Lincova E, Pernicova Z, et al. TGF-beta1 suppresses IL-6-induced STAT3 activation through regulation of Jak2 expression in prostate epithelial cells. Cell Signal, 2010, 22(11): 1734-1744.
[105] Weber A, Wasiliew P, Kracht M. Interleukin-1 (IL-1) pathway. Science Signaling, 2010, 3(105): 1-11.
[106] Cogswell J P, Godlevski M M, Wisely G B, et al. NF-kappa B regulates IL-1 beta transcription through a consensus NF-kappa B binding site and a nonconsensus CRE-like site. Journal of Immunology, 1994, 53(2): 712-723.
[107] Colotta F, Re F, Muzio M, et al. Interleukin-1 type II receptor: a decoy target for IL-1 that is regulated by IL-4. Science, 1993, 261(5120): 472-475.
[108] Holtmann H, Winzen R, Holland P, et al. Induction of Interleukin-8 synthesis integrates effects on transcription and mRNA degradation from at least three different cytokine- or stress-activated signal transduction pathways. Molecular and Cellular Biology, 1999, 19(10): 6742-6753.
[109] Walker P R, Saas P, Dietrich P Y. Role of Fas ligand (CD95L) in immune escape: The tumor cell strikes back. The Journal of Immunology, 1997, 158(10): 4521-4524.
[110] Hu X, Paik P K, Chen J, et al. IFN-gamma suppresses IL-10 production and synergizes with TLR2 by regulating GSK3 and CREB/AP-1 proteins. Immunity, 2006, 24(5): 563-574.
[111] Ponzetto C, Bardelli A, Maina F, et al. A novel recognition motif for phosphatidylinositol 3-kinase binding mediates its association with the hepatocyte growth factor/scatter factor receptor. Molecular and Cellular Biology, 1993, 13(8): 4600-4608.
[112] Ferrara N, Gerber H P, LeCouter J. The biology of VEGF and its receptors. Nature Medicine, 2003,9(6): 669-676.
[113] Avizienyte E, Wyke A W, Jones R J, et al. Src-induced de-regulation of E-cadherin in colon cancer cells requires integrin signalling. Nature Cell Biology, 2002, 4(8): 632-638.
[114] Rommel C, Bodine S C, Clarke B A, et al. Mediation of IGF-1-induced skeletal myotube hypertrophy by PI(3)K/Akt/mTOR and PI(3)K/Akt/GSK3 pathways. Nature Cell Biology, 2001, 3(11): 1009-1013.
[115] Castellano E, Downward J. RAS Interaction with PI3K: More Than Just Another Effector Pathway. Genes & Cancer, 2011, 2(3): 261-274.
[116] Leslie N R, Batty I H, Maccario H, et al. Understanding PTEN regulation: PIP2, polarity and protein stability. Oncogene, 2008, 27(41): 5464-5476.
[117] Krymskaya V P, Hoffman R, Eszterhas A, et al. TGF-beta 1 modulates EGF-stimulated phosphatidylinositol 3-kinase activity in human airway smooth muscle cells. The American Journal of Physiology, 1997, 273(6): L1220-1227.
[118] Waugh D J J, Wilson C. The Interleukin-8 pathway in cancer. Clinical Cancer Research, 2008, 14(21): 6735-6741.
[119] Gustafsson M V, Zheng X W, Pereira T, et al. Hypoxia requires notch signaling to maintain the undifferentiated cell state. Development Cell, 2005, 9(5): 617-628.
[120] Vousden K H, Evan G I. Proliferation, cell cycle and apoptosis in cancer[J]. Nature, 2001, 411(6835): 342-348.
[121] Ayer D E, Eisenman R N. A switch from Myc:Max to Mad:Max heterocomplexes accompanies monocyte/macrophage differentiation. Genes & development, 1993, 7(11): 2110-2119.
[122] Manning B D, Cantley L C. AKT/PKB signaling: Navigating downstream. Cell, 2007, 129(7): 1261-1274.
[123] Vasudevan K M, Gurumurthy S, Rangnekar V M. Suppression of PTEN expression by NF-κB prevents apoptosis. Molecular and Cellular Biology, 2004, 24(3): 1007-1021.
[124] Chagpar R B, Links P H, Pastor M C, et al. Direct positive regulation of PTEN by the p85 subunit of phosphatidylinositol 3-kinase. Proceedings of the National Academy of Sciences of the United States of America, 2010, 107(12): 5471-2476.
[125] Lau M T, Klausen C, Leung P C. E-cadherin inhibits tumor cell growth by suppressing PI3K/Akt signaling via beta-catenin-Egr1-mediated PTEN expression. Oncogene, 2011, 30(24): 2753-2766.
[126] Finch A, Holland P, Cooper J, et al. Selective activation of JNK/SAPK by interleukin-1 in rabbit liver is mediated by MKK7. Federation of European Biochemical Societies Letters, 1997, 418(1-2): 144-148.
[127] Stupack D G, Cheresh D A. Get a ligand, get a life: Integrins, signaling and cell survival. Journal of Cell Science, 2002, 115(19):

3729-3738.
[128] Chalhoub N, Baker S J. PTEN and the PI3-kinase pathway in cancer. Annual Review of Pathology, 2009, 4: 127-150.
[129] Engel M E, McDonnell M A, Law B K, et al. Interdependent SMAD and JNK signaling in transforming growth factor-β-mediated transcription. Journal of Biological Chemistry, 1999, 274(52): 37413-37420.
[130] Adler V, Pincus M R, Brandt-Rauf P W, et al. Complexes of p21RAS with JUN N-terminal kinase and JUN proteins[C]. Proceedings of the National Academy of Sciences, 1995, 92(23): 10585-10589.
[131] Kanakaraj P, Schafer P H, Cavender D E, et al. Interleukin (IL)-1 receptor-associated kinase (IRAK) requirement for optimal induction of multiple IL-1 signaling pathways and IL-6 production. The Journal of Experimental Medicine, 1998, 187(12): 2073-2079.
[132] Waite K A, Eng C. Protean PTEN: Form and function. The American Journal of Human Genetics, 2002, 70(4): 829-844.
[133] Chi H, Barry S P, Roth R J, et al. Dynamic regulation of proand anti-inflammatory cytokines by MAPK phosphatase 1 (MKP-1) in innate immune responses[C]. Proceedings of the National Academy of Sciences, 2006, 103(7): 2274-2279.
[134] Ferrara N, Gerber H P, LeCouter J. The biology of VEGF and its receptors. Nature Medicine, 2003,9(6): 669-676.
[135] Hu J H, Chen T, Zhuang Z H, et al. Feedback control of MKP-1 expression by p38. Cell Signal, 2007, 19(2): 393-400.
[136] Wang G L, Semenza G L. General involvement of hypoxia-inducible factor 1 in transcriptional response to hypoxia. Proceedings of the National Academy of Sciences of the United States of America, 1993, 90(9): 4304-4308.
[137] Wei D Y, Le X D, Zheng L Z, et al. Stat3 activation regulates the expression of vascular endothelial growth factor and human pancreatic cancer angiogenesis and metastasis. Oncogene, 2003, 22(3): 319-329.
[138] Wu T. Cyclooxygenase-2 in hepatocellular carcinoma. Cancer Treatment Reviews, 2006, 32(1): 28-44.
[139] Hatakeyama M, Imaizumi T, Sakaki H, et al. Interleukin-1 induces the expression of vascular endothelial growth factor in human pericardial mesothelial cells. Heart and Vessels, 2007, 22(2): 123-127.
[140] Lu J M, Zhang Z Z, Ma X, et al. Repression of microRNA-21 inhibits retinal vascular endothelial cell growth and angiogenesis via PTEN dependent-PI3K/Akt/VEGF signaling pathway in diabetic retinopathy. Experimental Eye Research, 2020, 190: 107886.
[141] Chang Y J, Wu M S, Lin J T, et al. Helicobacter pylori-Induced invasion and angiogenesis of gastric cells is mediated by cyclooxygenase-2 induction through TLR2/TLR9 and promoter regulation. The Journal of Immunology, 2005, 175(12): 8242-8252.
[142] Ho H L, Kao H L, Yeh Y C, et al. The importance of EGFR mutation testing in squamous cell carcinoma or non-small cell carcinoma favor squamous cell carcinoma diagnosed from small lung biopsies. Diagnostic Pathology, 2019, 14(1): 1-8.
[143] Tzahar E, Waterman H, Chen X, et al. A hierarchical network of interreceptor interactions determines signal transduction by Neu differentiation factor/neuregulin and epidermal growth factor. Molecular and Cellular Biology, 1996, 16(10): 5276-5287.
[144] Ohlsson C, Kley N, Werner H, et al. p53 regulates insulin-like growth factor-I (IGF-I) receptor expression and IGF-I-induced tyrosine phosphorylation in an osteosarcoma cell line: interaction between p53 and Sp1. Endocrinology, 1998, 139(3): 1101-1107.
[145] Gambarotta G, Boccaccio C, Giordano S, et al. Ets up-regulates MET transcription. Search Life-sciences Literature, 1996, 13(9): 1911-1917.
[146] Pennacchietti S, Michieli P, Galluzzo M, et al. Hypoxia promotes invasive growth by transcriptional activation of the met protooncogene. Cancer Cell, 2003, 3(4): 347-361.
[147] Boccaccio C, Comoglio P M. Invasive growth: a MET-driven genetic programme for cancer and stem cells. Nature Reviews Cancer, 2006, 6(8): 637-645.
[148] Pabst T, Mueller B U, Harakawa N, et al. AML1-ETO downregulates the granulocytic differentiation factor C/EBPalpha in t(8;21) myeloid leukemia. Nature Medicine, 2001, 7(4): 444-451.
[149] Ross S E, Erickson R L, Hemati N, et al. Glycogen synthase kinase 3 is an insulin-regulated C/EBPalpha kinase. Molecular and Cellular Biology, 1999, 19(12): 8433-8441.
[150] An M R, Hsieh C C, Reisner P D, et al. Evidence for posttranscriptional regulation of C/EBPalpha and C/EBPbeta isoform expression during the lipopolysaccharide-mediated acute-phase response. Molecular and Cellular Biology, 1996, 16(5): 2295-2306.
[151] Rosen E D, MacDougald O A. Adipocyte differentiation from the inside out. Nature Reviews Molecular Cell Biology, 2006, 7(12): 885-896.
[152] Chan P C, Chen S Y, Chen C H, et al. Crosstalk between hepatocyte growth factor and integrin signaling pathways. Journal of Biomedical Science, 2006, 13(2): 215-223.
[153] Chan A O O, Huang C, Hui W M, et al. Stability of E-cadherin methylation status in gastric mucosa associated with histology changes. Alimentary Pharmacology & Therapeutics, 2006, 24(5): 831-836.
[154] Steinhusen U, Weiske J, Badock V, et al. Cleavage and shedding of E-cadherin after induction of apoptosis. Journal of Biological

Chemistry, 2001, 276(7): 4972-4980.

[155] Laux H, Tomer R, Mader M T, et al. Tumor-associated E-cadherin mutations do not induce Wnt target gene expression, but affect E-cadherin repressors. Laboratory Investigation; A Journal of Technical Methods and Pathology, 2004, 84(10): 1372-1386.

[156] Seo H, Amano T, Seki R, et al. Upstream enhancer elements of Shh regulate oral and dental patterning. Journal of Dental Research, 2018, 97(9): 1055-1063.

[157] Danilkovitch-Miagkova A, Miagkov A, Skeel A, et al. Oncogenic mutants of RON and MET receptor tyrosine kinases cause activation of the beta-catenin pathway. Molecular and Cellular Biology, 2001, 21(17): 5857-5868.

[158] Nelson W J, Nusse R. Convergence of Wnt, beta-catenin, and cadherin pathways. Science, 2004, 303(5663): 1483-1487.

[159] Morali O G, Delmas V, Moore R, et al. IGF-II induces rapid beta-catenin relocation to the nucleus during epithelium to mesenchyme transition. Oncogene, 2001, 20(36): 4942-4950.

[160] Brigidi G S, Bamji S X. Cadherin-catenin adhesion complexes at the synapse. Current Opinion in Neurobiology. 2011, 21(2): 208-214.

[161] Niehrs C. The complex world of WNT receptor signalling. Nature Reviews Molecular Cell Biology, 2012, 13(12): 767-779.

[162] Cherukuri P, DeCastro A J, Balboni A L, et al. Phosphorylation of ΔNp63α via a novel TGFβ/ALK5 signaling mechanism mediates the anti-clonogenic effects of TGFβ. Public Library of Science one, 2012, 7(11): e50066.

[163] Sundqvist A, Vasilaki E, Voytyuk O, et al. TGFβ and EGF signaling orchestrates the AP-1- and p63 transcriptional regulation of breast cancer invasiveness. Oncogene, 2020, 39(22): 4436-4449.

[164] Ho J Y, Chang F W, Huang F S, et al. Estrogen enhances the cell viability and motility of breast cancer cells through the ERα-ΔNp63-Integrin β4 signaling pathway. Public Library of Science one, 2016, 11(2): e0148301.

[165] Ye S, Lee K B, Park M H, et al. p63 regulates growth of esophageal squamous Carcinoma cells via the Akt signaling pathway. International Journal of Oncology, 2014, 44(6): 2153-2159.

[166] Shimura T, Noma N, Oikawa T, et al. Activation of the AKT/cyclin D1/Cdk4 survival signaling pathway in radioresistant cancer stem cells. Oncogenesis, 2012, 1(6): e12.

[167] Meng F, Liu L, Chin P C. Akt is a downstream target of NF-kappa B. Journal of Biological Chemistry, 2002, 277(33): 29674-29680.

[168] Kong X, Zhao Y, Li X, et al. Overexpression of HIF-2alpha-dependent NEAT1 promotes the progression of non-small cell lung cancer through miR-101- 3p/SOX9/Wnt/beta-catenin signal pathway. Cell Physiol Biochem, 2019, 52(3): 368- 381.

[169] Furumatsu T, Tsuda M, Taniguchi N, et al. Smad3 induces chondrogenesis through the activation of SOX9 via CREB-binding protein/p300 recruitment. Journal of Biological Chemistry, 2005, 280(9): 8343-8350.

[170] Huang J Q, Wei F K, Xu X L, et al. SOX9 drives the epithelial-mesenchymal transition in non-small-cell lung cancer through the Wnt/β-catenin pathway. Journal of Translational Medicine, 2019, 17(1): 143.

[171] Murakami S, Lefebvre V, Crombrugghe D B. Potent inhibition of the master chondrogenic factor Sox9 gene by Interleukin-1 and tumor necrosis factor-α. Journal of Biological Chemistry, 2000, 275(5): 3687-3692.

[172] Maier J A, Lo Y T, Harfe B D. Foxa1 and Foxa2 are required for formation of the intervertebral discs. Public Library of Science one, 2013, 8(1): e55528.

[173] Zhang X, Lou Y, Wang H, et al. Wnt signaling regulates the stemness of lung cancer stem cells and its inhibitors exert anticancer effect on lung cancer SPC-A1 cells. Medical Oncology, 2015, 32(4): 1-8.

[174] Zhuang X, Zhang H, Li X, et al. Differential effects on lung and bone metastasis of breast cancer by Wnt signalling inhibitor DKK1. Nature Cell Biology, 2017, 19(10): 1274-1285.

[175] Sinha A, Fan V B, Ramakrishnan A B, et al. Repression of Wnt/β-catenin signaling by SOX9 and Mastermind-like transcriptional coactivator 2. Science Advances, 2021, 7(8): eabe0849.

[176] Song B, Park S H, Zhao J C, et al. Targeting FOXA1-mediated repression of TGF-β signaling suppresses castration-resistant prostate cancer progression. The Journal of Clinical Investigation. 2019, 129(2): 569-582.

[177] Kouros-Mehr H, Slorach E M, Sternlicht, et al. GATA-3 maintains the differentiation of the luminal cell fate in the mammary gland. Cell, 2006, 127(5): 1041-1055.

[178] Theodorou V, Stark R, Menon S, et al. GATA3 acts upstream of FOXA1 in mediating ESR1 binding by shaping enhancer accessibility. Genome Research, 2013, 23(1): 12-22.

[179] Márquez D C, Lee J, Lin T, et al. Epidermal growth factor receptor and tyrosine phosphorylation of estrogen receptor. Endocrine, 2001, 16(2): 73-81.

[180] Medunjanin S, Hermani A, Servi B D, et al. Glycogen synthase kinase-3 interacts with and phosphorylates estrogen receptor alpha and is involved in the regulation of receptor activity.

[181] Cheng J W, Zhang C, Shapiro D J. A functional serine 118 phosphorylation site in estrogen receptor-alpha is required for down-regulation of gene expression by 17beta-estradiol and 4-hydroxytamoxifen. Endocrinology, 2007, 148(10): 4634-4641.
[182] Eeckhoute J, Keeton E K, Lupien M, et al. Positive cross-regulatory loop ties GATA-3 to estrogen receptor alpha expression in breast cancer. Cancer Research, 2007, 67(13): 6477-6483. The Journal of Biological Chemistry, 2005, 280(38): 33006-33014.
[183] Yang Z B, Barnes C J, Kumar R. Human epidermal growth factor receptor 2 status modulates subcellular localization of and interaction with estrogen receptor alpha in breast cancer cells. Clinical Cancer Research, 2004, 10(11): 3621-3618.
[184] Ballaré C, Uhrig M, Bechtold T, et al. Two domains of the progesterone receptor interact with the estrogen receptor and are required for progesterone activation of the c-Src/Erk pathway in mammalian cells. Molecular and Cellular Biology, 2003, 23(6): 1994-2008.
[185] Storci G, Sansone P, Mari S, et al. TNFalpha up-regulates SLUG via the NF-kappaB/HIF1alpha axis, which imparts breast cancer cells with a stem cell-like phenotype. Journal of Cellular Physiology, 2010, 225(3): 682-691.
[186] Izzo F, Mercogliano F, Venturutti L, et al. Progesterone receptor activation downregulates GATA3 by transcriptional repression and increased protein turnover promoting breast tumor growth. Breast Cancer Research, 2014, 16(6): 491.
[187] Panet-Raymond V, Gottlieb B, Beitel L K, et al. Interactions between androgen and estrogen receptors and the effects on their transactivational properties. Molecular and Cellular Endocrinology, 2000, 167(1-2): 139-150.
[188] Lange C A, Shen T, Horwitz K B. Phosphorylation of human progesterone receptors at serine-294 by mitogen-activated protein kinase signals their degradation by the 26S proteasome. roceedings of the National Academy of Sciences of the United States of America, 2000, 97(3):1032-1037.
[189] Deb T B, Su L, Wong L, et al. Epidermal growth factor (EGF) receptor kinase-independent signaling by EGF. The Journal of Biological Chemistry, 2001, 276(18): 15554-15560.
[190] Patten B A, Sardi S P, Koirala S, et al. Notch1 signaling regulates radial glia differentiation through multiple transcriptional mechanisms. The Journal of Neuroscience, 2006, 26(12): 3102-3108.
[191] Musgrove E A. Cyclins: roles in mitogenic signaling and oncogenic transformation. Growth Factors, 2006, 24(1):13-19.
[192] Das J, Chen C H, Yang L, et al. A critical role for NF-kappa B in GATA3 expression and TH2 differentiation in allergic airway inflammation. Nature Immunology, 2001, 2(1): 45-50.
[193] Takaku M, Grimm S A, Kumar B D, et al. Cancer-specific mutation of GATA3 disrupts the transcriptional regulatory network governed by Estrogen Receptor alpha, FOXA1 and GATA3. Nucleic Acids Research, 2020, 48(9): 4756-4768.
[194] Liang Y, Xu Y, Lu B, et al. Inositol Alleviates Pulmonary Fibrosis by Promoting Autophagy via Inhibiting the HIF-1 α-SLUG Axis in Acute Respiratory Distress Syndrome. Oxidative Medicine and Cellular Longevity. 2022, 2022: 1030238.
[195] Zhang C, Carl T F, Trudeau E D, et al. An NF-kappaB and slug regulatory loop active in early vertebrate mesoderm. Library of Science one, 2006, 1(1): e106.
[196] Wang Y F, Shi J, Chai K, et al. The role of Snail in EMT and tumorigenesis. Current Cancer Drug Targets, 2013, 13(9): 963-972.
[197] Son H, Moon A. Epithelial-mesenchymal transition and cell invasion. Toxicological Research, 2010, 26(4): 245-252.
[198] Zhou B P, Deng J, Xia W, et al. Dual regulation of Snail by GSK-3beta-mediated phosphorylation in control of epithelial-mesenchymal transition. Nature Cell Biology, 2004, 6(10): 931-940.
[199] Kim J Y, Kim Y M, Yang C H, et al. Functional regulation of Slug/Snail2 is dependent on GSK-3β-mediated phosphorylation. Federation of European Biochemical Societies Journal ,2012, 279(16): 2929-2939.
[200] Ye Y, Xiao Y, Wang W, et al. ERalpha suppresses slug expression directly by transcriptional repression. The Biochemical Journal, 2008, 416(2): 179-187.
[201] Jiang N, Zou C, Zhu Y, et al. HIF-1α-regulated miR-1275 maintains stem cell-like phenotypes and promotes the progression of LUAD by simultaneously activating Wnt/β-catenin and Notch signaling. Theranostics, 2020, 10(6): 2553-2570.
[202] Johnson E A. HIF takes it up a notch. Science Signaling, 2011, 4(181): pe33.
[203] Deshmukh A P, Vasaikar S V, Tomczak K, et al. Identification of EMT signaling cross-talk and gene regulatory networks by single-cell RNA sequencing. Proceedings of the National Academy of Sciences of the United States of America, 2021, 118(19): e2102050118.
[204] Sakamoto K, Chao W S, Katsube K, et al. Distinct roles of EGF repeats for the Notch signaling system. Experimental Cell Research 2005, 302(2): 281-291.
[205] Shi Y C, Zhao H, Ying C, et al. C/EBPα inhibits hepatocellular carcinoma by reducing Notch3/Hes1/p27 cascades. Digestive and Liver Disease 2013, 45(10): 844-851.
[206] Rizzo P, Miao H, G D'Souza, et al. Cross-talk between notch and the estrogen receptor in breast cancer suggests novel therapeutic approaches. Cancer Research, 2008, 68(13):5226-5235.

B. Ordinary differential equations (ODEs) generated from Table S1. We took $n_i = 3, a_i = 8$ for all the factors.

List S2. ODEs for the endogenous network

$$\frac{dx_1}{dt} = \frac{1}{1 + a_1 \cdot x_2^{n_1} + a_2 \cdot x_3^{n_2} + a_3 \cdot x_9^{n_3}} - x_1$$

$$\frac{dx_2}{dt} = \frac{a_4 \cdot x_8^{n_4} + a_5 \cdot x_{21}^{n_5} + a_6 \cdot x_{32}^{n_6} + a_7 \cdot x_{33}^{n_7} + a_8 \cdot x_{43}^{n_8}}{1 + a_4 \cdot x_8^{n_4} + a_5 \cdot x_{21}^{n_5} + a_6 \cdot x_{32}^{n_6} + a_7 \cdot x_{33}^{n_7} + a_8 \cdot x_{43}^{n_8}} \cdot \frac{1}{a_9 \cdot x_7^{n_9} + a_{10} \cdot x_{45}^{n_{10}}} - x_2$$

$$\frac{dx_3}{dt} = \frac{a_{11} \cdot x_4^{n_{11}} + a_{12} \cdot x_8^{n_{12}}}{1 + a_{11} \cdot x_4^{n_{11}} + a_{12} \cdot x_8^{n_{12}}} \cdot \frac{1}{a_{13} \cdot x_7^{n_{13}} + a_{14} \cdot x_6^{n_{14}}} - x_3$$

$$\frac{dx_4}{dt} = \frac{a_{15} \cdot x_8^{n_{15}} + a_{16} \cdot x_4^{n_{16}}}{1 + a_{15} \cdot x_8^{n_{15}} + a_{16} \cdot x_4^{n_{16}}} \cdot \frac{1}{a_{17} \cdot x_1^{n_{17}} + a_{18} \cdot x_7^{n_{18}}} - x_4$$

$$\frac{dx_5}{dt} = \frac{a_{19} \cdot x_{37}^{n_{19}} + a_{20} \cdot x_{39}^{n_{20}} + a_{21} \cdot x_{41}^{n_{21}} a_{22} \cdot x_{35}^{n_{22}}}{1 + a_{19} \cdot x_{37}^{n_{19}} + a_{20} \cdot x_{39}^{n_{20}} + a_{21} \cdot x_{41}^{n_{21}} a_{22} \cdot x_{35}^{n_{22}}} - x_5$$

$$\frac{dx_6}{dt} = \frac{a_{23} \cdot x_{20}^{n_{23}} + a_{24} \cdot x_{31}^{n_{24}} + a_{25} \cdot x_{42}^{n_{25}} + a_{26} \cdot x_{19}^{n_{26}}}{1 + a_{23} \cdot x_{20}^{n_{23}} + a_{24} \cdot x_{31}^{n_{24}} + a_{25} \cdot x_{42}^{n_{25}} + a_{26} \cdot x_{19}^{n_{26}}} \cdot \frac{1}{a_{27} \cdot x_8^{n_{27}} + a_{28} \cdot x_{29}^{n_{28}} + a_{29} \cdot x_{32}^{n_{29}} + a_{30} \cdot x_3^{n_{30}}} - x_6$$

$$\frac{dx_7}{dt} = \frac{a_{31} \cdot x_{22}^{n_{31}} + a_{32} \cdot x_{19}^{n_{32}} + a_{33} \cdot x_{20}^{n_{33}} + a_{34} \cdot x_{30}^{n_{34}} + a_{35} \cdot x_{32}^{n_{35}} + a_{36} \cdot x_{50}^{n_{36}} + a_{37} \cdot x_4^{n_{37}}}{1 + a_{31} \cdot x_{22}^{n_{31}} + a_{32} \cdot x_{19}^{n_{32}} + a_{33} \cdot x_{20}^{n_{33}} + a_{34} \cdot x_{30}^{n_{34}} + a_{35} \cdot x_{32}^{n_{35}} + a_{36} \cdot x_{50}^{n_{36}} + a_{37} \cdot x_4^{n_{37}}} \cdot \frac{1}{a_{38} \cdot x_8^{n_{38}} + a_{39} \cdot x_{29}^{n_{39}}} - x_7$$

$$\frac{dx_8}{dt} = \frac{a_{40} \cdot x_{29}^{n_{40}} + a_{41} \cdot x_{21}^{n_{41}} + a_{42} \cdot x_{30}^{n_{42}} + a_{43} \cdot x_{32}^{n_{43}} + a_{44} \cdot x_{33}^{n_{44}} + a_{45} \cdot x_{43}^{n_{45}} + a_{46} \cdot x_4^{n_{46}}}{1 + a_{40} \cdot x_{29}^{n_{40}} + a_{41} \cdot x_{21}^{n_{41}} + a_{42} \cdot x_{30}^{n_{42}} + a_{43} \cdot x_{32}^{n_{43}} + a_{44} \cdot x_{33}^{n_{44}} + a_{45} \cdot x_{43}^{n_{45}} + a_{46} \cdot x_4^{n_{46}}} \cdot \frac{1}{a_{47} \cdot x_{19}^{n_{47}} + a_{48} \cdot x_{20}^{n_{48}} + a_{49} \cdot x_{22}^{n_{49}} + a_{50} \cdot x_7^{n_{50}}} - x_8$$

$$\frac{dx_9}{dt} = \frac{a_{51} \cdot x_{12}^{n_{51}} + a_{52} \cdot x_{18}^{n_{52}} + a_{53} \cdot x_{10}^{n_{53}}}{1 + a_{50} \cdot x_{10}^{n_{50}} + a_{51} \cdot x_{12}^{n_{51}} + a_{52} \cdot x_{18}^{n_{52}}} \cdot \frac{1}{a_{54} \cdot x_{15}^{n_{54}}} - x_9$$

$$\frac{dx_{10}}{dt} = \frac{a_{55} \cdot x_4^{n_{55}} + a_{56} \cdot x_{11}^{n_{56}} + a_{57} \cdot x_{12}^{n_{57}} + a_{58} \cdot x_{13}^{n_{58}}}{1 + a_{55} \cdot x_4^{n_{55}} + a_{56} \cdot x_{11}^{n_{56}} + a_{57} \cdot x_{12}^{n_{57}} + a_{58} \cdot x_{13}^{n_{58}}} \cdot \frac{1}{a_{59} \cdot x_{15}^{n_{59}} + a_{60} \cdot x_{29}^{n_{60}}} - x_{10}$$

$$\frac{dx_{11}}{dt} = \frac{a_{61} \cdot x_{18}^{n_{61}}}{1 + a_{61} \cdot x_{18}^{n_{61}}} \cdot \frac{1}{a_{62} \cdot x_{15}^{n_{62}}} - x_{11}$$

$$\frac{dx_{12}}{dt} = \frac{a_{63} \cdot x_{22}^{n_{63}}}{1 + a_{63} \cdot x_{22}^{n_{63}}} \cdot \frac{1}{a_{64} \cdot x_{29}^{n_{64}} + a_{65} \cdot x_{32}^{n_{65}}} - x_{12}$$

$$\frac{dx_{13}}{dt} = \frac{a_{66} \cdot x_8^{n_{66}} + a_{67} \cdot x_{22}^{n_{67}}}{1 + a_{66} \cdot x_8^{n_{66}} + a_{67} \cdot x_{22}^{n_{67}}} \cdot \frac{1}{a_{68} \cdot x_{16}^{n_{68}} + a_{69} \cdot x_{17}^{n_{69}}} - x_{13}$$

$$\frac{dx_{14}}{dt} = \frac{a_{70} \cdot x_{19}^{n_{70}} + a_{71} \cdot x_{21}^{n_{71}} + a_{72} \cdot x_{22}^{n_{72}}}{1 + a_{70} \cdot x_{19}^{n_{70}} + a_{71} \cdot x_{21}^{n_{71}} + a_{72} \cdot x_{22}^{n_{72}}} \cdot \frac{1}{a_{73} \cdot x_5^{n_{73}}} - x_{14}$$

$$\frac{dx_{15}}{dt} = \frac{a_{74} \cdot x_{21}^{n_{74}} + a_{75} \cdot x_{29}^{n_{75}}}{1 + a_{74} \cdot x_{21}^{n_{74}} + a_{75} \cdot x_{29}^{n_{75}}} \cdot \frac{1}{a_{76} \cdot x_{10}^{n_{76}}} - x_{15}$$

$$\frac{dx_{16}}{dt} = \frac{a_{77} \cdot x_{21}^{n_{77}} + a_{78} \cdot x_{32}^{n_{78}} + a_{79} \cdot x_{35}^{n_{79}} + a_{80} \cdot x_{55}^{n_{80}}}{1 + a_{77} \cdot x_{21}^{n_{77}} + a_{78} \cdot x_{32}^{n_{78}} + a_{79} \cdot x_{35}^{n_{79}} + a_{80} \cdot x_{55}^{n_{80}}} \cdot \frac{1}{a_{81} \cdot x_8^{n_{81}} + a_{82} \cdot x_{12}^{n_{82}} + a_{83} \cdot x_{22}^{n_{83}} + a_{84} \cdot x_{49}^{n_{84}}} - x_{16}$$

$$\frac{dx_{17}}{dt} = \frac{a_{85} \cdot x_{21}^{n_{85}} + a_{86} \cdot x_{37}^{n_{86}} + a_{87} \cdot x_{38}^{n_{87}}}{1 + a_{85} \cdot x_{21}^{n_{85}} + a_{86} \cdot x_{37}^{n_{86}} + a_{87} \cdot x_{38}^{n_{87}}} \cdot \frac{1}{a_{88} \cdot x_8^{n_{88}} + a_{89} \cdot x_{12}^{n_{89}}} - x_{17}$$

$$\frac{dx_{18}}{dt} = \frac{a_{90} \cdot x_{14}^{n_{90}} + a_{91} \cdot x_{24}^{n_{91}} + a_{92} \cdot x_{26}^{n_{92}}}{1 + a_{90} \cdot x_{14}^{n_{90}} + a_{91} \cdot x_{24}^{n_{91}} + a_{92} \cdot x_{26}^{n_{92}}} - x_{18}$$

$$\frac{dx_{19}}{dt} = \frac{a_{93} \cdot x_{26}^{n_{93}}}{1 + a_{93} \cdot x_{26}^{n_{93}}} \cdot \frac{1}{a_{94} \cdot x_{28}^{n_{94}}} - x_{19}$$

$$\frac{dx_{20}}{dt} = \frac{a_{95} \cdot x_{30}^{n_{95}} + a_{96} \cdot x_{41}^{n_{96}} + a_{97} \cdot x_{46}^{n_{97}}}{1 + a_{95} \cdot x_{30}^{n_{95}} + a_{96} \cdot x_{41}^{n_{96}} + a_{97} \cdot x_{46}^{n_{97}}} \cdot \frac{1}{a_{98} \cdot x_{21}^{n_{98}}} - x_{20}$$

$$\frac{dx_{21}}{dt} = \frac{a_{99} \cdot x_{33}^{n_{99}} + a_{100} \cdot x_{44}^{n_{100}}}{1 + a_{99} \cdot x_{33}^{n_{99}} + a_{100} \cdot x_{44}^{n_{100}}} \cdot \frac{1}{a_{101} \cdot x_{23}^{n_{101}} + a_{102} \cdot x_{25}^{n_{102}}} - x_{21}$$

$$\frac{dx_{22}}{dt} = \frac{a_{103} \cdot x_{4}^{n_{103}} + a_{104} \cdot x_{8}^{n_{104}} + a_{105} \cdot x_{10}^{n_{105}} + a_{106} \cdot x_{21}^{n_{106}}}{1 + a_{103} \cdot x_{4}^{n_{103}} + a_{104} \cdot x_{8}^{n_{104}} + a_{105} \cdot x_{10}^{n_{105}} + a_{106} \cdot x_{21}^{n_{106}}} \cdot \frac{1}{a_{107} \cdot x_{29}^{n_{107}}} - x_{22}$$

$$\frac{dx_{23}}{dt} = \frac{a_{108} \cdot x_{21}^{n_{108}} + a_{109} \cdot x_{32}^{n_{109}} + a_{110} \cdot x_{33}^{n_{110}}}{1 + a_{108} \cdot x_{21}^{n_{108}} + a_{109} \cdot x_{32}^{n_{109}} + a_{110} \cdot x_{33}^{n_{110}}} \cdot \frac{1}{a_{111} \cdot x_{24}^{n_{111}} + a_{112} \cdot x_{14}^{n_{112}} + a_{113} \cdot x_{20}^{n_{113}} + a_{114} \cdot x_{26}^{n_{114}} + a_{115} \cdot x_{29}^{n_{115}}} - x_{23}$$

$$\frac{dx_{24}}{dt} = \frac{a_{116} \cdot x_{21}^{n_{116}} + a_{117} \cdot x_{32}^{n_{117}} + a_{118} \cdot x_{33}^{n_{118}}}{1 + a_{116} \cdot x_{21}^{n_{116}} + a_{117} \cdot x_{32}^{n_{117}} + a_{118} \cdot x_{33}^{n_{118}}} \cdot \frac{1}{a_{119} \cdot x_{28}^{n_{119}}} - x_{24}$$

$$\frac{dx_{25}}{dt} = \frac{a_{120} \cdot x_{28}^{n_{120}} + a_{121} \cdot x_{37}^{n_{121}} + a_{122} \cdot x_{47}^{n_{122}} + a_{123} \cdot x_{39}^{n_{123}}}{1 + a_{120} \cdot x_{28}^{n_{120}} + a_{121} \cdot x_{37}^{n_{121}} + a_{122} \cdot x_{47}^{n_{122}} + a_{123} \cdot x_{39}^{n_{123}}} \cdot \frac{1}{a_{124} \cdot x_{20}^{n_{124}}} - x_{25}$$

$$\frac{dx_{26}}{dt} = \frac{a_{125} \cdot x_{21}^{n_{125}} + a_{126} \cdot x_{33}^{n_{126}}}{1 + a_{125} \cdot x_{21}^{n_{125}} + a_{126} \cdot x_{33}^{n_{126}}} \cdot \frac{1}{a_{127} \cdot x_{26}^{n_{127}}} - x_{26}$$

$$\frac{dx_{27}}{dt} = \frac{a_{128} \cdot x_{21}^{n_{128}} + a_{129} \cdot x_{33}^{n_{129}}}{1 + a_{128} \cdot x_{21}^{n_{128}} + a_{129} \cdot x_{33}^{n_{129}}} - x_{27}$$

$$\frac{dx_{28}}{dt} = \frac{a_{130} \cdot x_{14}^{n_{130}} + a_{131} \cdot x_{24}^{n_{131}}}{1 + a_{130} \cdot x_{14}^{n_{130}} + a_{131} \cdot x_{24}^{n_{131}}} \cdot \frac{1}{a_{132} \cdot x_{19}^{n_{132}}} - x_{28}$$

$$\frac{dx_{29}}{dt} = \frac{a_{133} \cdot x_{5}^{n_{133}} + a_{134} \cdot x_{39}^{n_{134}} + a_{135} \cdot x_{27}^{n_{135}} + a_{136} \cdot x_{35}^{n_{136}} + a_{137} \cdot x_{37}^{n_{137}} + a_{138} \cdot x_{38}^{n_{138}} + a_{139} \cdot x_{20}^{n_{139}} + a_{140} \cdot x_{41}^{n_{140}} + a_{141} \cdot x_{42}^{n_{141}}}{1 + a_{133} \cdot x_{5}^{n_{133}} + a_{134} \cdot x_{20}^{n_{134}} + a_{135} \cdot x_{27}^{n_{135}} + a_{136} \cdot x_{35}^{n_{136}} + a_{137} \cdot x_{37}^{n_{137}} + a_{138} \cdot x_{38}^{n_{138}} + a_{139} \cdot x_{39}^{n_{139}} + a_{140} \cdot x_{41}^{n_{140}} + a_{141} \cdot x_{42}^{n_{141}}} \cdot \frac{1}{a_{142} \cdot x_{31}^{n_{142}}} - x_{29}$$

$$\frac{dx_{30}}{dt} = \frac{a_{143} \cdot x_{8}^{n_{143}} + a_{144} \cdot x_{55}^{n_{144}} + a_{145} \cdot x_{29}^{n_{145}}}{1 + a_{143} \cdot x_{8}^{n_{143}} + a_{144} \cdot x_{55}^{n_{144}} + a_{145} \cdot x_{29}^{n_{145}}} \cdot \frac{1}{a_{146} \cdot x_{22}^{n_{146}}} - x_{30}$$

$$\frac{dx_{31}}{dt} = \frac{1}{+ a_{147} \cdot x_{21}^{n_{147}} + a_{148} \cdot x_{29}^{n_{148}} + a_{149} \cdot x_{43}^{n_{149}}} - x_{31}$$

$$\frac{dx_{32}}{dt} = \frac{a_{150} \cdot x_{5}^{n_{150}} + a_{151} \cdot x_{41}^{n_{151}} + a_{152} \cdot x_{25}^{n_{152}} + a_{153} \cdot x_{47}^{n_{153}}}{1 + a_{150} \cdot x_{5}^{n_{150}} + a_{151} \cdot x_{41}^{n_{151}} + a_{152} \cdot x_{25}^{n_{152}} + a_{153} \cdot x_{47}^{n_{153}}} \cdot \frac{1}{a_{154} \cdot x_{31}^{n_{154}} + a_{155} \cdot x_{34}^{n_{155}}} - x_{32}$$

$$\frac{dx_{33}}{dt} = \frac{a_{156} \cdot x_{20}^{n_{156}} + a_{157} \cdot x_{24}^{n_{157}} + a_{158} \cdot x_{26}^{n_{158}} + a_{159} \cdot x_{27}^{n_{159}} + a_{160} \cdot x_{35}^{n_{160}} + a_{161} \cdot x_{37}^{n_{161}} + a_{162} \cdot x_{47}^{n_{162}}}{1 + a_{156} \cdot x_{20}^{n_{156}} + a_{157} \cdot x_{24}^{n_{157}} + a_{158} \cdot x_{26}^{n_{158}} + a_{159} \cdot x_{27}^{n_{159}} + a_{160} \cdot x_{35}^{n_{160}} + a_{161} \cdot x_{37}^{n_{161}} + a_{162} \cdot x_{47}^{n_{162}}} \cdot \frac{1}{a_{163} \cdot x_{31}^{n_{163}} + a_{164} \cdot x_{34}^{n_{164}}} - x_{33}$$

$$\frac{dx_{34}}{dt} = \frac{a_{165} \cdot x_{32}^{n_{165}} + a_{166} \cdot x_{33}^{n_{166}}}{1 + a_{165} \cdot x_{32}^{n_{165}} + a_{166} \cdot x_{33}^{n_{166}}} - x_{34}$$

$$\frac{dx_{35}}{dt} = \frac{a_{167} \cdot x_{30}^{n_{167}} + a_{168} \cdot x_{25}^{n_{168}} + a_{169} \cdot x_{27}^{n_{169}} + a_{170} \cdot x_{36}^{n_{170}} + a_{171} \cdot x_{39}^{n_{171}} + a_{172} \cdot x_{26}^{n_{172}} + a_{173} \cdot x_{29}^{n_{173}} + a_{174} \cdot x_{41}^{n_{174}}}{1 + a_{167} \cdot x_{30}^{n_{167}} + a_{168} \cdot x_{25}^{n_{168}} + a_{169} \cdot x_{27}^{n_{169}} + a_{170} \cdot x_{36}^{n_{170}} + a_{171} \cdot x_{39}^{n_{171}} + a_{172} \cdot x_{26}^{n_{172}} + a_{173} \cdot x_{29}^{n_{173}} + a_{174} \cdot x_{41}^{n_{174}}} - x_{35}$$

$$\frac{dx_{36}}{dt} = \frac{a_{175} \cdot x_{21}^{n_{175}} + a_{176} \cdot x_{33}^{n_{176}}}{1 + a_{175} \cdot x_{21}^{n_{175}} + a_{176} \cdot x_{33}^{n_{176}}} - x_{36}$$

$$\frac{dx_{37}}{dt}=\frac{a_{177}\cdot x_{27}^{n_{177}}+a_{178}\cdot x_{41}^{n_{178}}+a_{179}\cdot x_{44}^{n_{179}}+a_{180}\cdot x_{52}^{n_{180}}}{1+a_{177}\cdot x_{27}^{n_{177}}+a_{178}\cdot x_{41}^{n_{178}}+a_{179}\cdot x_{44}^{n_{179}}+a_{180}\cdot x_{52}^{n_{180}}}-x_{37}$$

$$\frac{dx_{38}}{dt}=\frac{a_{181}\cdot x_{40}^{n_{181}}}{1+a_{181}\cdot x_{40}^{n_{181}}}\cdot\frac{1}{a_{182}\cdot x_{22}^{n_{182}}}-x_{38}$$

$$\frac{dx_{39}}{dt}=\frac{a_{183}\cdot x_{32}^{n_{183}}+a_{184}\cdot x_{30}^{n_{184}}+a_{185}\cdot x_{43}^{n_{185}}}{1+a_{183}\cdot x_{32}^{n_{183}}+a_{184}\cdot x_{30}^{n_{184}}+a_{185}\cdot x_{43}^{n_{185}}}-x_{39}$$

$$\frac{dx_{40}}{dt}=\frac{a_{186}\cdot x_{40}^{n_{186}}+a_{187}\cdot x_{45}^{n_{187}}}{1+a_{186}\cdot x_{40}^{n_{186}}+a_{187}\cdot x_{45}^{n_{187}}}\cdot\frac{1}{a_{188}\cdot x_{26}^{n_{188}}+a_{189}\cdot x_{53}^{n_{189}}}-x_{40}$$

$$\frac{dx_{41}}{dt}=\frac{a_{190}\cdot x_{37}^{n_{190}}+a_{191}\cdot x_{39}^{n_{191}}}{1+a_{190}\cdot x_{37}^{n_{190}}+a_{191}\cdot x_{39}^{n_{191}}}\cdot\frac{1}{a_{192}\cdot x_{31}^{n_{192}}}-x_{41}$$

$$\frac{dx_{42}}{dt}=\frac{1}{a_{193}\cdot x_{26}^{n_{193}}+a_{194}\cdot x_{39}^{n_{194}}+a_{195}\cdot x_{9}^{n_{195}}+a_{196}\cdot x_{41}^{n_{196}}+a_{197}\cdot x_{54}^{n_{197}}}-x_{42}$$

$$\frac{dx_{43}}{dt}=\frac{a_{198}\cdot x_{47}^{n_{198}}+a_{199}\cdot x_{37}^{n_{199}}+a_{200}\cdot x_{39}^{n_{200}}+a_{201}\cdot x_{38}^{n_{201}}}{1+aa_{198}\cdot x_{47}^{n_{198}}+a_{199}\cdot x_{37}^{n_{199}}+a_{200}\cdot x_{39}^{n_{200}}+a_{201}\cdot x_{38}^{n_{201}}}\cdot\frac{1}{a_{202}\cdot x_{42}^{n_{202}}+a_{203}\cdot x_{45}^{n_{203}}}-x_{43}$$

$$\frac{dx_{44}}{dt}=\frac{a_{204}\cdot x_{20}^{n_{204}}+a_{205}\cdot x_{37}^{n_{205}}}{1+a_{204}\cdot x_{20}^{n_{204}}+a_{205}\cdot x_{37}^{n_{205}}}\cdot\frac{1}{a_{206}\cdot x_{29}^{n_{206}}+a_{207}\cdot x_{50}^{n_{207}}}-x_{44}$$

$$\frac{dx_{45}}{dt}=\frac{1}{a_{208}\cdot x_{21}^{n_{208}}+a_{209}\cdot x_{29}^{n_{209}}}-x_{45}$$

$$\frac{dx_{46}}{dt}=\frac{a_{210}\cdot x_{20}^{n_{210}}+a_{211}\cdot x_{30}^{n_{211}}+a_{212}\cdot x_{48}^{n_{212}}}{1+a_{210}\cdot x_{20}^{n_{210}}+a_{211}\cdot x_{30}^{n_{211}}+a_{212}\cdot x_{48}^{n_{212}}}\cdot\frac{1}{a_{213}\cdot x_{21}^{n_{213}}+a_{214}\cdot x_{26}^{n_{214}}}-x_{46}$$

$$\frac{dx_{47}}{dt}=\frac{a_{215}\cdot x_{43}^{n_{215}}+a_{216}\cdot x_{49}^{n_{216}}}{1+a_{215}\cdot x_{43}^{n_{215}}+a_{216}\cdot x_{49}^{n_{216}}}-x_{47}$$

$$\frac{dx_{48}}{dt}=\frac{a_{217}\cdot x_{20}^{n_{217}}+a_{218}\cdot x_{37}^{n_{218}}}{1+a_{217}\cdot x_{20}^{n_{217}}+a_{218}\cdot x_{37}^{n_{218}}}\cdot\frac{1}{a_{219}\cdot x_{45}^{n_{219}}+a_{220}\cdot x_{46}^{n_{220}}}-x_{48}$$

$$\frac{dx_{49}}{dt}=\frac{a_{221}\cdot x_{20}^{n_{221}}+a_{222}\cdot x_{53}^{n_{222}}}{1+a_{221}\cdot x_{20}^{n_{221}}+a_{222}\cdot x_{53}^{n_{222}}}-x_{49}$$

$$\frac{dx_{50}}{dt}=\frac{a_{223}\cdot x_{37}^{n_{223}}+a_{224}\cdot x_{45}^{n_{224}}+a_{225}\cdot x_{32}^{n_{225}}+a_{226}\cdot x_{51}^{n_{226}}+a_{227}\cdot x_{52}^{n_{227}}+a_{228}\cdot x_{53}^{n_{228}}}{1+a_{223}\cdot x_{37}^{n_{223}}+a_{224}\cdot x_{45}^{n_{224}}+a_{225}\cdot x_{32}^{n_{225}}+a_{226}\cdot x_{51}^{n_{226}}+a_{227}\cdot x_{52}^{n_{227}}+a_{228}\cdot x_{53}^{n_{228}}}\cdot\frac{1}{a_{229}\cdot x_{54}^{n_{229}}}-x_{50}$$

$$\frac{dx_{51}}{dt}=\frac{a_{230}\cdot x_{53}^{n_{230}}}{1+a_{230}\cdot x_{53}^{n_{230}}}\cdot\frac{1}{a_{231}\cdot x_{32}^{n_{231}}+a_{232}\cdot x_{50}^{n_{232}}}-x_{51}$$

$$\frac{dx_{52}}{dt}=\frac{a_{233}\cdot x_{37}^{n_{233}}+a_{234}\cdot x_{55}^{n_{234}}}{1+a_{233}\cdot x_{37}^{n_{233}}+a_{234}\cdot x_{55}^{n_{234}}}\cdot\frac{1}{a_{235}\cdot x_{3}^{n_{235}}}-x_{52}$$

$$\frac{dx_{53}}{dt}=\frac{a_{236}\cdot x_{21}^{n_{236}}+a_{237}\cdot x_{50}^{n_{237}}}{1+aa_{236}\cdot x_{21}^{n_{236}}+a_{237}\cdot x_{50}^{n_{237}}}\cdot\frac{1}{a_{238}\cdot x_{16}^{n_{238}}}-x_{53}$$

$$\frac{dx_{54}}{dt} = \frac{a_{239} \cdot x_{20}^{n_{339}} + a_{240} \cdot x_{21}^{n_{240}} + a_{241} \cdot x_{30}^{n_{241}} + a_{242} \cdot x_{55}^{n_{242}}}{1 + a_{239} \cdot x_{20}^{n_{339}} + a_{240} \cdot x_{21}^{n_{240}} + a_{241} \cdot x_{30}^{n_{241}} + a_{242} \cdot x_{55}^{n_{242}}} \cdot \frac{1}{a_{243} \cdot x_{45}^{n_{243}} + a_{244} \cdot x_{50}^{n_{244}}} - x_{54}$$

$$\frac{dx_{55}}{dt} = \frac{a_{245} \cdot x_{20}^{n_{245}} + a_{246} \cdot x_{30}^{n_{246}} + a_{247} \cdot x_{37}^{n_{247}}}{1 + a_{245} \cdot x_{20}^{n_{245}} + a_{246} \cdot x_{30}^{n_{246}} + a_{247} \cdot x_{37}^{n_{247}}} \cdot \frac{1}{a_{248} \cdot x_{40}^{n_{248}} + a_{249} \cdot x_{50}^{n_{249}}} - x_{55}.$$

C. Relative expression levels of the molecular factors clustered by modules, under different steady states. The levels then set the ON/OFF switch of the modules where they reside.

Table S2. Expression of module factors and switch vs. steady states

**States**	S1	S2	S3	S4	S5	S6	S7	S8	S9	S10	S11	S12	S13
Rb	0.16	0.10	0.10	0.16	0.14	0.13	0.14	1.00	1.00	0.14	1.00	1.00	1.00
CyclinD-CDK4,6	0.87	0.87	0.87	0.87	0.00	0.00	0.00	0.00	0.00	0.00	0.00	0.00	0.00
CyclinE-CDK2	0.11	0.79	0.79	0.11	0.00	0.00	0.00	0.00	0.00	0.00	0.00	0.00	0.00
E2F	0.11	0.78	0.79	0.10	0.00	0.00	0.00	0.00	0.00	0.00	0.00	0.00	0.00
Ras	0.96	0.96	0.96	0.96	0.90	0.00	0.81	0.00	0.89	0.81	0.81	0.90	0.81
p27	0.11	0.11	0.11	0.11	0.91	0.93	0.91	0.91	0.86	0.93	0.91	0.87	0.86
p21	0.24	0.24	0.24	0.24	0.00	0.00	0.00	0.00	0.00	0.00	0.00	0.01	0.00
Myc	0.08	0.06	0.06	0.08	0.87	0.91	0.87	0.94	0.92	0.90	0.93	0.92	0.92
Cell cycle	OFF	ON	OFF	ON	OFF	OFF	OFF	OFF	OFF	OFF	OFF	OFF	OFF
caspase3	0.08	0.08	0.08	0.08	0.91	0.94	0.91	0.00	0.00	0.91	0.00	0.00	0.00
Cytochrome c	0.00	0.06	0.06	0.00	0.88	0.93	0.89	0.00	0.00	0.88	0.00	0.00	0.00
Bid	0.04	0.10	0.10	0.04	0.84	0.86	0.84	0.00	0.00	0.84	0.00	0.00	0.00
Bad	0.08	0.08	0.08	0.08	0.00	0.82	0.00	0.00	0.00	0.00	0.00	0.00	0.00
Bax	0.00	0.00	0.00	0.00	0.81	0.82	0.81	0.00	0.00	0.81	0.00	0.00	0.00
Fas	0.02	0.02	0.02	0.02	0.80	0.83	0.83	0.00	0.00	0.80	0.00	0.00	0.00
XIAP	0.03	0.03	0.03	0.03	0.12	0.83	0.16	0.00	0.00	0.16	0.00	0.00	0.00
Bcl-2	0.89	0.88	0.88	0.89	0.00	0.00	0.00	0.01	0.01	0.00	0.01	0.01	0.01
Bcl-xL	0.21	0.13	0.21	0.13	0.06	0.00	0.06	0.00	0.81	0.00	0.00	0.15	0.15
caspase8,10	0.83	0.82	0.82	0.83	0.16	0.00	0.00	0.00	0.81	0.15	0.81	0.81	0.00
Cell apoptosis	OFF	OFF	OFF	OFF	ON	ON	ON	OFF	OFF	ON	OFF	OFF	OFF
IFN-γ	0.50	0.49	0.49	0.49	0.01	0.82	0.03	0.00	0.00	0.03	0.00	0.00	0.00
TGFb	0.19	0.19	0.19	0.19	0.00	0.00	0.00	0.00	0.00	0.00	0.00	0.00	0.00
NF-kB	0.71	0.71	0.71	0.71	0.01	0.81	0.00	0.81	0.01	0.81	0.81	0.01	0.00
p53	0.34	0.34	0.34	0.34	0.00	0.02	0.00	0.02	0.00	0.02	0.02	0.00	0.00
ikB	0.05	0.05	0.05	0.05	0.02	0.00	0.02	0.00	0.02	0.00	0.00	0.02	0.02
TNFa	0.43	0.43	0.43	0.43	0.02	0.00	0.02	0.02	0.02	0.02	0.02	0.02	0.02
STAT3	0.25	0.24	0.25	0.24	0.90	0.17	0.82	0.16	0.81	0.17	0.17	0.90	0.81
IL-1	0.35	0.35	0.35	0.35	0.01	0.01	0.01	0.01	0.01	0.01	0.01	0.01	0.01
IL-8	0.48	0.47	0.48	0.48	0.01	0.01	0.01	0.01	0.01	0.01	0.01	0.01	0.01
IL-10	0.37	0.37	0.37	0.37	0.01	0.82	0.03	0.00	0.00	0.03	0.00	0.00	0.00
Inflammatory	ON	ON	ON	ON	OFF	OFF	OFF	OFF	OFF	OFF	OFF	OFF	OFF
PI3K-Akt	0.97	0.97	0.97	0.97	0.11	0.09	0.10	0.11	0.11	0.11	0.11	0.11	0.11
HIF	0.89	0.88	0.88	0.89	0.00	0.00	0.00	0.01	0.01	0.00	0.01	0.01	0.01
PTEN	0.06	0.06	0.06	0.06	0.98	0.99	0.98	0.99	0.99	0.98	0.99	0.99	0.99
Erk1/2	0.42	0.42	0.42	0.42	0.11	0.10	0.11	0.10	0.10	0.11	0.10	0.11	0.11
SAPK	0.42	0.42	0.42	0.42	0.11	0.10	0.10	0.10	0.10	0.11	0.11	0.11	0.10
MKP	0.54	0.54	0.54	0.54	0.02	0.02	0.02	0.02	0.02	0.02	0.02	0.02	0.02

Metabolism	ON	ON	ON	ON	OFF	OFF	OFF	OFF	OFF	OFF	OFF	OFF	OFF
VEGF	0.97	0.97	0.97	0.97	0.85	0.05	0.81	0.04	0.81	0.05	0.05	0.85	0.81
COX-2	0.48	0.47	0.48	0.48	0.01	0.01	0.01	0.01	0.01	0.01	0.01	0.01	0.01
EGF	0.93	0.88	0.88	0.92	0.81	0.01	0.00	0.01	0.81	0.81	0.81	0.81	0.00
IGF	0.85	0.84	0.84	0.85	0.00	0.00	0.00	0.00	0.00	0.00	0.00	0.00	0.00
HGF	0.93	0.93	0.93	0.93	0.02	0.01	0.02	0.01	0.01	0.02	0.01	0.01	0.01
CEBPa	0.01	0.00	0.01	0.00	0.14	0.14	0.14	0.14	0.90	0.14	0.14	0.15	0.15
Angiogenesis	ON	ON	ON	ON	OFF	OFF	OFF	OFF	OFF	OFF	OFF	OFF	OFF
Integrin	0.93	0.92	0.92	0.93	0.09	0.00	0.00	0.00	0.09	0.09	0.09	0.09	0.00
E-cadherin	0.05	0.07	0.05	0.07	0.14	0.13	0.14	1.00	0.99	0.14	0.99	0.99	1.00
**States**	S1	S2	S3	S4	S5	S6	S7	S8	S9	S10	S11	S12	S13
b-catenin	0.95	0.94	0.95	0.95	0.10	0.09	0.09	0.05	0.05	0.10	0.05	0.05	0.05
p63	0.11	0.06	0.11	0.06	0.10	0.11	0.00	0.11	0.11	0.11	0.11	0.10	0.00
GSK3B	0.11	0.12	0.12	0.11	0.99	0.99	0.99	0.99	0.99	0.99	0.99	0.99	0.99
SOX9	0.54	0.54	0.54	0.54	0.00	0.81	0.00	0.81	0.00	0.81	0.81	0.00	0.00
SHH	0.91	0.92	0.91	0.93	0.83	0.86	0.82	0.86	0.00	0.86	0.86	0.82	0.82
WNT	0.40	0.39	0.39	0.40	0.09	0.06	0.00	0.06	0.09	0.07	0.07	0.09	0.00
Cell adhesion	OFF	OFF	OFF	OFF	OFF	OFF	OFF	ON	ON	OFF	ON	ON	ON
Cell state	C	C	C	C	A	A	A	N	N	A	N	N	N

D. The expression levels of breast cancer specific (BCS) modules under simulated alternations to the endogenous network.

Table S3. Expression of BCS factors under simulations

BCS Factors	Block the inhibition from Slug to ER		Block the inhibition from ER to Slug		Knocked out Slug	
	State1	State2	State1	State2	State1	State2
ER	0.9583	0.9372	0.1044	0.1106	0.9372	0.9583
PR	0.0846	0.0892	0.0958	0.0971	0.0892	0.0846
HER2	0.8847	0.121	0.1288	0.9337	0.121	0.8847
GATA3	0.8914	0.8855	0.261	0.2625	0.8855	0.8914
Slug	0.0936	0.0993	0.9098	0.9107	-	-
Notch	0.097	0.1026	0.9369	0.938	0.1026	0.097
Foxa1	0.9126	0.9116	0.7782	0.7783	0.9116	0.9126
